# Stratification of telomerase activity in cancer reveals associations with senescence and genomic instability

**DOI:** 10.1016/j.csbj.2025.11.020

**Published:** 2025-11-14

**Authors:** Nighat Noureen, Min Hee Kang

**Affiliations:** Cancer Center and Pediatrics, School of Medicine, Texas Tech University Health Sciences Center, Lubbock, TX, USA

**Keywords:** Telomerase, Senescence, Inflammation, Unsupervised learning, Pan-cancer, Single-cell, Spatial transcriptomics

## Abstract

Telomerase activity plays an essential role in tumor growth and varies across cancers, typically classified as low or high based on its expression level. This variation is pertinent to cancer-related mechanisms and hallmarks of cellular aging. However, the relationship between distinct telomerase activity groups (low or high) and specific molecular programs across tumor types remains poorly defined, largely due to the absence of a robust classification framework. Here, we applied EXTEND, our previously validated computational model for quantifying telomerase activity, to stratify tumors into low and high telomerase activity groups across diverse cancer types using an unsupervised, data-driven approach. We analyzed over 10,000 tumor samples from bulk RNA sequencing data in The Cancer Genome Atlas (TCGA) and the Cancer Cell Line Encyclopedia (CCLE), as well as more than 10,000 single cells from single-cell and spatial transcriptomic datasets. Our analyses revealed that high telomerase activity group was strongly associated with genomic instability across majority of cancers, whereas low telomerase activity group was enriched for cellular senescence, inflammation, reactive oxygen species (ROS), and MAPK signaling pathways. Notably, cellular senescence, a hallmark of aging, was predominant in older individuals across cancers, normal tissues, and developmental stages. Together, our findings establish a comprehensive framework linking telomerase activity groups to distinct molecular and cellular phenotypes across human cancers and reveal that low telomerase activity corresponds to a senescence-like transcriptional program that is generally associated with favorable survival outcomes. Conclusively, our work provides a unifying framework for understanding telomerase-associated heterogeneity across a broad compendium of tumors.

## Highlights

• Unsupervised classification of telomerase activity.
• Distribution of telomerase activity levels across Pan cancer at bulk, single and spatial resolution.
• Modulation of telomerase levels by genomic instability.
• Association of telomerase activity levels with cellular senescence across different cancer histologies.
• Immune inflammation association with low telomerase activity across pan cancer.

## Graphical Abstract

**Figure:**
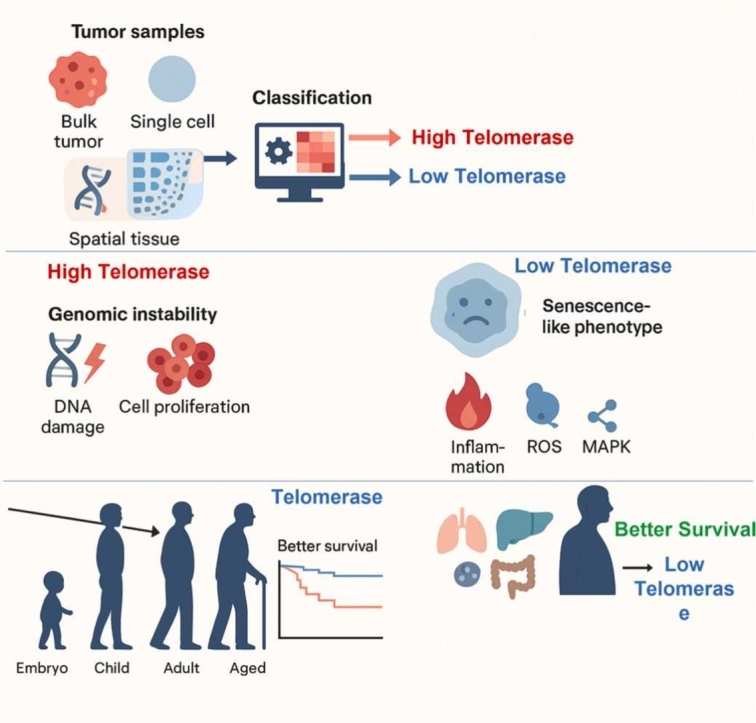

## 1 Background

Telomeres are nucleoproteins containing canonical repeats (TTAGGG) at the ends of chromosomes that play a vital role in maintaining the integrity of the linear chromosomes during cell division and prevent them from being recognized as double-strand breaks [1]. Generally, the length of telomeres varies from 4 to 10 kilobases and protects the DNA erosion due to the ‘end-replication problem’ - which is defined as the lack of ability of DNA polymerase to fully replicate the terminal DNA sequences [2]. Telomeres progressively shorten due to end replication problems by each cell division [3] due to oxidative stress and inflammation that cause DNA damage [4]. Persistent shortening and dysfunction of telomeres impact cellular functions, thereby leading to senescence and cell death [5]. On the contrary, the maintenance of telomere lengths enables bypassing senescence, thereby leading to immortality [6]. To achieve replicative immortality, cancer cells must maintain their telomeres so that they do not shorten upon each cell division, thereby counteracting the end replication problem. The ability to enable replicative immortality by activating a telomere maintenance mechanism (TMM) is one of the hallmarks of cancer [7].

Approximately 85–90 % of cancer cells use the ribonucleoprotein, telomerase, containing a catalytic subunit (*TERT*) and an RNA template (*TERC*) to add sequential TTAGGG telomeric repeats to the ends of chromosomes [8], [9], [10]. The RNA subunit of telomerase, *TERC* is ubiquitously expressed across tissues [11], [12]. The catalytic subunit *TERT* is expressed in most cancers and proliferating cells. Cancer cells activate telomerase to gain immortality by acquiring *TERT* promoter mutations and rearrangements, or transcriptional dysregulation [13], [14]. *TERT* promoter mutations augment the transcriptional output of *TERT* [15], [16], [17], [18] and, in certain cases, correlate with increased *TERT* expression and telomerase activity [19], [20]. Though *TERT* and *TERC* are major telomerase subunits, in many cases, *TERT* alone contributes to telomerase activity and is considered a limiting factor for it [13], [14], [15], [16], [18], [21], [22], [23], [24]. Nonetheless, we and others have reported that *TERT* expression is not a reliable measure of telomerase activity under different scenarios [12], [25], [26], [27], [28], [29], [30], [31], [32]. Recent quantification of telomerase enzymatic activities using EXTEND [29] has shown that cancer types exhibit a broad range of activity levels, where both low and high telomerase activities across different cancer histologies are associated with patient prognosis.

Although majorly silent or extremely low in somatic cells, telomerase is active in stem cells, rapidly generating mitotically active cells [9], [33] and is reactivated in the majority of human cancers [34]. Telomerase levels are heterogeneous across cancers and tissue types [34], [35], [36], [37], [38]. This heterogeneity is associated with distinct tumor stages [39], [40], molecular subtypes [29], and several signaling pathways. Growing evidence indicates non-canonical functions of telomerase that affect various cellular processes, including signaling, regulation of cell survival, resistance to stress, and apoptosis [41]. It also affects cancer invasiveness and metastasis by WNT and NF-kB signaling mechanisms [42], [43]. The effect of telomerase on diverse signaling pathways largely depends on its expression levels across tumors from different tissues. Tumor cells with high telomerase activity are highly proliferative and exhibit enhanced self-renewal capacity, thereby promoting tumor growth. In contrast, tumors with low telomerase activity display reduced proliferative potential. Nonetheless, varying levels of telomerase activity can elicit distinct signaling cascades that substantially influence cellular processes such as proliferation, senescence and apoptosis [44], [45], [46].

We and others have previously analyzed telomere maintenance mechanisms, *TERT* expression, and telomere length across various cancers [29], [47], [48], [49]. However, telomerase activity levels have not yet been systematically classified or analyzed at a pan-cancer scale. In this study, we apply an unsupervised data-driven approach to stratify tumors into low and high telomerase activity groups across a comprehensive collection of datasets, including bulk tissue, single-cell, and spatial transcriptomic profiles. This analytical framework builds upon our previously established telomerase activity quantification method, EXTEND [29], to evaluate telomerase activity across both cancerous and normal tissues. Our integrative analysis reveals distinct associations between telomerase activity groups and key biological processes, including senescence, immune inflammation, and genome instability across diverse tumor types. Notably, tumors with lower levels of telomerase activities exhibit senescence-associated phenotypes, which is a suitable candidate for favorable survival outcomes.

## 2 Results

### 2.1 Unsupervised classification of telomerase activity

To characterize telomerase activity across human cancers, we analyzed quantitative telomerase activity scores generated by EXTEND (EXpression-based Telomerase ENzymatic activity Detection) for 33 cancer types in The Cancer Genome Atlas (TCGA), as reported previously [29].Tumor-associated normal samples (n = 700) were excluded from analysis. The analyzed TCGA cancer types included adrenocortical carcinoma (ACC), bladder urothelial carcinoma (BLCA), breast invasive carcinoma (BRCA), cervical squamous cell carcinoma and endocervical adenocarcinoma (CESC), cholangiocarcinoma (CHOL), colon adenocarcinoma (COAD), diffuse large B-cell lymphoma (DLBC), esophageal carcinoma (ESCA), glioblastoma multiforme (GBM), head and neck squamous cell carcinoma (HNSC), kidney chromophobe (KICH), kidney renal clear cell carcinoma (KIRC), kidney renal papillary cell carcinoma (KIRP), acute myeloid leukemia (LAML), brain lower-grade glioma (LGG), liver hepatocellular carcinoma (LIHC), lung adenocarcinoma (LUAD), lung squamous cell carcinoma (LUSC), mesothelioma (MESO), ovarian serous cystadenocarcinoma (OV), pancreatic adenocarcinoma (PAAD), pheochromocytoma and paraganglioma (PCPG), prostate adenocarcinoma (PRAD), rectum adenocarcinoma (READ), sarcoma (SARC), skin cutaneous melanoma (SKCM), stomach adenocarcinoma (STAD), testicular germ cell tumors (TGCT), thymoma (THYM), thyroid carcinoma (THCA), uterine corpus endometrial carcinoma (UCEC), uterine carcinosarcoma (UCS), and uveal melanoma (UVM). Hereafter, cancer types are referred to by their TCGA abbreviations.

**Fig. 1:**
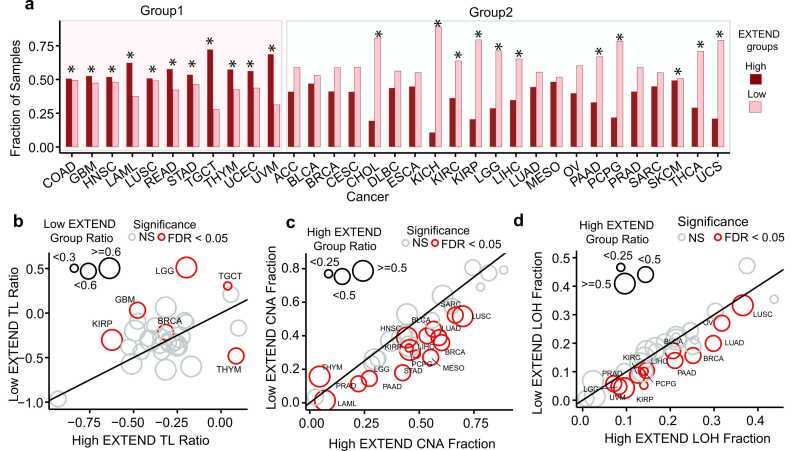
Differences between low and high telomerase activity groups. (a) Distribution of low (pink) and high (dark red) telomerase activity (EXTEND) groups across 33 TCGA cancer types. Cancer types are classified into Group 1 (predominantly high telomerase activity) or Group 2 (predominantly low telomerase activity). X-axis represents cancer types and Y-axis represents proportion of samples across 33 cancers for low and high telomerase activity groups. Statistically significant differences (FDR < 0.05; Fisher’s exact test) are indicated by an asterisk (*). (b-d) Comparisons between low and high telomerase activity groups for (b) telomere length (TL) ratios (c) copy number altered (CNA) fractions, and (d) loss of heterozygosity (LOH) fractions. Cancer types with significant differences (FDR < 0.05; Student’s *t*-test) are labeled and highlighted in red; non-significant (NS) cancer types are shown in grey. Circle size corresponds to the proportion of samples in low telomerase activity group for b, and in high telomerase activity group for c and d. X-axes represent TL, CNA, and LOH ratios/fractions in high telomerase activity groups, while Y-axes represent those in low telomerase activity groups. Source data are provided in GitHub repository.

Within each cancer type, we stratified tumors into low and high telomerase activity groups using unsupervised *k*-means [50] clustering (*k* = 2) of EXTEND scores. We ensured clustering robustness through 1000 iterations across 50 random initializations, followed by consensus clustering of 50,000 partitions. This approach produced stable group assignments. Overall, 57 % of TCGA tumors were classified as low and 43 % as high telomerase activity. Distinct trends emerged across cancer types. Eleven of the 33 cancer types showed significant enrichment for the high telomerase activity group (FDR < 0.05; Fisher’s exact test; Group 1; average odds ratio=1.93), including gastrointestinal (COAD, READ, STAD), reproductive (UCEC, TGCT), and several others (LUSC, HNSC, THYM, UVM, and, LAML; Fig. 1a). Notably, TGCT displayed the strongest enrichment, with 72 % of tumors assigned to the high telomerase activity group — approximately threefold more frequent than low telomerase activity group (n = 28 %). In contrast, the remaining 22 cancer types (Group 2) including KICH, KIRC, KIRP, LGG, THCA, PCPG, and PAAD, exhibited the opposite trend, with a significantly higher proportion of tumors classified as low telomerase activity (FDR < 0.05; Fisher’s exact test; average odds ratio = 0.6). The proportion of tumors in each activity group for all cancer types is summarized in Fig. 1 source data.

### 2.2 Association of clinical features with low and high telomerase activity groups

To identify clinical and epidemiological factors associated with telomerase activity, we analyzed TCGA tumors classified into low and high telomerase activity groups. Of the 33 cancer types analyzed, we excluded sex-specific cancers (prostate, breast, ovarian, uterine and testicular) to ensure a balanced representation of both sexes, resulting in 27 cancer types for sex-based analysis. Across these cancers, we observed a significant enrichment (*P* < 0.05; Fisher’s exact test; average odds ratio = 2; Supplementary Fig.1a) of female patients in the low telomerase activity group for several cancer types including LUAD (59 % vs. 46 %), LAML (63 % vs. 36 %), SKCM (43 % vs. 33 %), and HNSC (31 % vs. 21 %) – percentages are reported in the same order (low vs. high telomerase activity groups) throughout this section. These findings suggest that sex may represent an important etiological factor influencing cancers with low telomerase activity. In contrast, STAD was the only cancer type exhibiting a higher proportion of male patients in the low telomerase activity group (71 % vs 61 %; odds ratio = 0.65). These patterns are consistent with previous studies [51], [52], [53], [54] reporting higher incidence and poor survival among males for these cancer types.

For age-associated comparisons, we excluded cancers with fewer than 15 samples in either age group (18–50 years = young adults [YA], and > 50 = older adults [OA]) to ensure sufficient representation. Among the remaining 28 cancer types, older adults were significantly enriched in the low telomerase activity group for LUAD (95 % vs. 89 %), THYM (90 % vs. 59 %), and OV (82 % vs. 68 %) (*P* < 0.05; Fisher’s exact test; average odds ratio = 0.4; Supplementary Fig.1b). Conversely, GBM (32 % vs. 17 %), THCA (62 % vs. 44 %), LGG (80 % vs. 63 %), and UVM (40 % vs. 15 %) showed higher frequencies of low telomerase activity in young adults (*P* < 0.05; Fisher’s exact test; average odds ratio = 2.7; Supplementary Fig.1b). These contrasting patterns underscore tissue-specific regulation of telomerase activity across age groups, consistent with previous studies [55], [56], [57].

Next, we analyzed 21 cancer types with available staging information for tumor stage comparisons. Lower stage tumors (Stages I-II) were significantly enriched (*P* < 0.05; Fisher’s exact test; average odds ratio = 3; Supplementary Fig.1c) in the low telomerase activity group for ACC, LUAD, THCA, KIRC, and KIRP. No cancer type showed enrichment of high-stage tumors (Stages III-IV) in the low telomerase activity group, consistent with previous literature [39], [58], [59].

Finally, we reassessed patient prognosis using unsupervised classification of EXTEND scores. High telomerase activity was associated with worse overall survival in ten cancer types: MESO, THCA, PAAD, PRAD, PCPG, KIRC, KIRP, KICH, SARC, and ACC (log-rank test; *P* < 0.05; 95 % CI; Supplementary Fig.1d). In contrast, STAD and THYM exhibited the opposite pattern, consistent with the previous analysis [29]. The observed associations were based on a univariate Cox regression analysis, which inherently does not account for potential confounders, including tissue-specific effects, clinical and demographic characteristics, and diverse genomic alterations.

### 2.3 Genomic disparities affecting telomerase activity levels in cancer

To investigate genomic disparities associated with telomerase activity, we compared telomere lengths (TL), copy number alterations (CNAs), loss of heterozygosity (LOH), whole genome doubling (WGD) [60], tumor mutation burden (TMB), recurrent gene mutations, and gene fusions between low and high telomerase activity groups across TCGA cancer types.

Among 32 TCGA cancers with available telomere length data, six showed significant differences between telomerase activity groups (FDR < 0.05; Wilcoxon rank-sum test; 95 % CI; Fig. 1b). Five cancers (TGCT, KIRP, GBM, LGG, and BRCA) displayed an inverse association between telomerase activity and TL, with the low telomerase activity group exhibiting longer telomeres. THYM was the only exception, where the low telomerase activity group was associated with shorter telomeres. These patterns align with previous observations that on average testis exhibits the longest telomeres among human tissues [36], and that brain tumors with longer telomeres often display the alternative lengthening of telomeres (ALT) phenotype [47], a telomerase-independent mechanism of telomere maintenance.

Analysis of CNAs revealed that fifteen of the 33 cancer types exhibited significantly higher CNAs in the high telomerase activity group (FDR < 0.05; Student’s *t*-test; 95 % CI; average effect size = 0.63; Fig. 1c). Lung cancers (LUAD, LUSC) displayed the most pronounced CNA elevation, in agreement with previous reports [47]. Thymoma (THYM) again represented an exception, with elevated CNAs in the low telomerase activity group (effect size = −0.55), aligning with its telomere length and survival patterns (Fig. 1b; Supplementary Fig.1d). Analysis of LOH profiles revealed a similar trend, where thirteen of the 33 cancer types showed significantly higher LOH levels in the high telomerase activity group (FDR < 0.05; Student’s *t*-test; 95 % CI; average effect size = 0.55; Fig. 1d). No cancer type exhibited LOH enrichment in the low telomerase activity group. Consistent with these findings, nine cancers (TGCT, LUAD, LUSC, BRCA, BLCA, SKCM, KIRP, PRAD, and ACC) showed a significant association between WGD and high telomerase activity (Fisher’s exact test; *P* < 0.02; 95 % CI; Supplementary Fig.2), supporting a link between telomerase activity and genomic instability. These findings are consistent with a previous study that used *TERT* expression as a surrogate for telomerase activity in adrenocortical carcinoma [61].

**Fig. 2:**
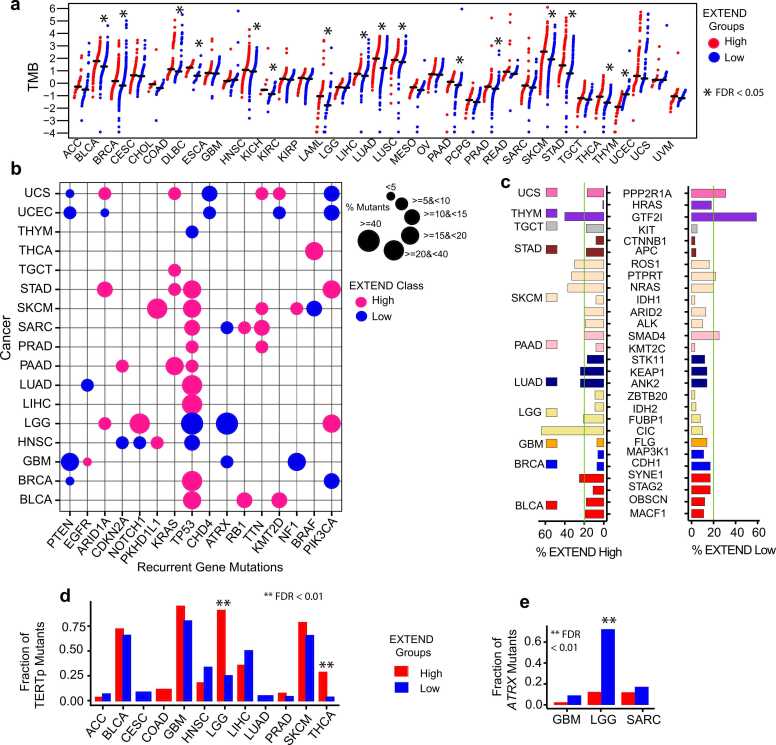
Mutational differences between low and high telomerase activity groups. (a) Tumor mutation burden (TMB on y-axis) across 33 cancer types (x-axis). Red indicates high and blue indicates low telomerase (EXTEND) activity groups. Statistically significant differences (FDR < 0.05; Wilcoxon rank-sum test) are marked with an asterisk (*) (b) Percentage of recurrent gene mutations (n = 16; x-axis) across 17 cancer types (y-axis) in high (pink) and low (blue) telomerase activity groups. Dot size represents the percentage of samples with each mutation, and color indicates telomerase activity group (c) Percentage of non-recurrent, cancer type-specific gene mutations (n = 28; x-axis) across 11 cancer types (y-axis) in low and high telomerase activity groups. Bar heights represent mutation frequency, and colors denote cancer types. (d-e) Differential distribution of (d) *TERT* promoter mutations (y-axis) across 12 cancer types (x-axis) and (e) *ATRX* mutations (y-axis) across three cancer types (x-axis). Significant differences between low (blue) and high (red) telomerase activity groups (FDR < 0.01; Fisher’s exact test) are indicated by an asterisk (*). Source data are provided in the GitHub repository.

Given the strong link between telomerase activity and genomic instability, we next compared TMB, an established aging-related feature [62] between low and high telomerase activity groups across 33 TCGA cancers. Sixteen cancer types showed significant differences, of which fifteen exhibited higher TMB in the high telomerase activity group (FDR < 0.05; Wilcoxon rank-sum test; 95 % CI; average Hodges-Lehmann estimate = 1.12; Fig. 2a). THYM was again the sole exception, with elevated TMB in the low telomerase activity group, consistent with its inverse survival pattern (Supplementary Fig.1d).

We next focused on cancers with significant TMB differences, to identify gene mutations associated with low and high telomerase activity groups. We identified 16 recurrent gene mutations (present in at least two cancers) across 17 cancers (Fig. 2b). Most recurrent gene mutations were enriched in the high telomerase activity group, consistent with their elevated TMB (Fig. 2a). *TP53* mutations were the most prevalent, observed in nine cancers for the high telomerase activity group but only three cancers (LGG, HNSC, and THYM) for the low telomerase activity group. Interestingly, *KRAS*, *PKHD1L1*, *RB1,* and *TTN* mutations were observed exclusively in the high telomerase activity groups, whereas *PTEN* and *ATRX* mutations were recurrent in low telomerase activity groups, consistent with ALT-associated phenotypes as reported previously [63].

Non-recurrent, cancer-specific mutations (n = 28 genes across 11 cancers; Fig. 2c) predominantly correlated with high telomerase activity, with notable exceptions including *PPP2R1A* in UCS, *HRAS,* and *GTF2I* in THYM and *SMAD4* in PAAD. The *CIC* gene mutations were significantly enriched in high telomerase activity LGG tumors, corresponding to high *TERT* expression (a surrogate of telomerase activity). This pattern mirrors the distribution of *TERT* promoter mutations, which were more frequent in high telomerase activity group in LGG (odds ratio = 28.5) and THCA (odds ratio = 11.6) (FDR < 0.01; Fisher’s exact test; 95 % CI; Fig. 2d). In contrast, *ATRX* gene mutations were enriched in low telomerase activity groups in LGG (104 in low vs. 7 in high), SARC (24-low vs. 13-high), and GBM (6-low vs. 2-high) (FDR < 0.01; Fisher’s exact test; 95 % CI; Fig. 2e) consistent with the presence of ALT phenotypes. Since ALT represents a telomerase-independent mechanism of telomere maintenance, we leveraged available annotations from LGG TCGA data [47] for ATRX-mutant (ALT or low telomerase activity) and *TERT* promoter mutant (high telomerase activity) cases to compare CNA’s, LOH, telomere length and telomerase activity scores between these two groups. We observed significantly higher CNA’s, LOH and telomerase activity patterns in *TERT* promoter mutant cases (high telomerase activity group), whereas ATRX mutant (ALT or low telomerase activity group) exhibited longer telomeres (Student’s *t*-test; *P* < 0.01; 95 % CI; Supplementary Figs.3a – 3d). These findings further support the enrichment of ALT-associated features in low telomerase activity group for LGG.

To investigate the mechanisms underlying the increased TMB and CNAs observed in high telomerase activity groups, we performed gene set pathway enrichment analysis across TCGA pan-cancer for low and high telomerase activity groups. Using pathways from Molecular Signatures Database [64], [65], the high telomerase activity group was predominantly associated with stemness and proliferation-related pathways. In contrast, the low telomerase activity group was enriched for muscle-associated pathways, reflecting features of differentiated skeletal muscle. Interestingly, MAPK target genes emerged as one of the top hits, showing upregulation in the low telomerase activity group (Supplementary Fig.4).

We next examined the association of telomerase activity groups with gene fusions, obtained from the TCGA study [66], which are critical drivers of oncogenesis. Overall, occurrences of fusion events were low across TCGA, with exceptions in seven cancer types (ACC, BRCA, KIRC, LUAD, OV, PRAD, and READ), where high telomerase activity groups exhibited significantly increased fusion frequencies (Student’s *t*-test; *P* < 0.05; 95 % CI; Supplementary Fig. 5a). Recurrent gene fusion analysis identified 18 cancer types with one or two recurrent fusions and four cancer types (ESCA, GBM, LAML, and PRAD) with > 2 recurrent fusions, with LAML showing the largest difference between low and high telomerase activity groups (5 vs.8; Supplementary Fig. 5b). Recurrent fusions were defined as those present in > 1 % of samples per cancer type.

**Fig. 3:**
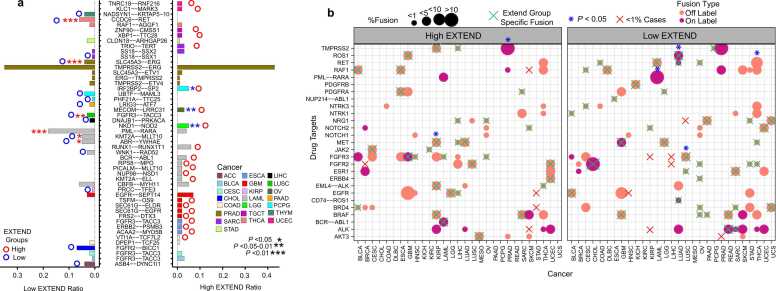
Variations in gene fusions between low and high telomerase activity groups. (a) Recurrent gene fusion fractions (x-axis) across TCGA pan-cancer for low and high telomerase activity (EXTEND) groups. Only fusions present in more than 1 % of cases per cancer type (y-axis) were included in the analysis. Bar plots show the ratio of fusions in low and high telomerase activity groups. Statistically significant differences (*P* < 0.05–0.01; Fisher’s exact test) are indicated by an asterisk (*). Blue (low) and red (high) circles above selected bars denote telomerase activity group-specific gene fusions. Bar colors represent individual cancer types (b) Association of druggable gene fusion targets with low and high telomerase activity groups. Drug targets (y-axis) occurring in more than 1 % of cases per cancer type (x-axis) were included. Asterisks (blue) denote significantly differential fusions between low and high telomerase groups (Fisher’s exact test, *P* < 0.05). Telomerase activity group-specific fusions are marked with a green cross (X) or shown in red when sample size is below 1 % for that group. Data point size reflects the percentage of fusion-positive cases. Point colors indicate fusion type(off-label or on-label). Source data are available in the GitHub repository.

Among these recurrent fusions, 22 were specific to the high telomerase activity group and 15 to the low telomerase activity group. Nine fusions showed distinct patterns, with three enriched in high and six in low telomerase activity groups. Significant fusions (*P* < 0.05; Fisher’s exact test; 95 % CI; Fig. 3a) associated with high telomerase activity group included IRF2BP2-SP2 (PCPG), MECOM-LRRC31(OV), and NKD1-NOD2(LGG), whereas those enriched in low telomerase activity group included PML-RARA, KMT2A-MLLT10, and ABR-YWHAE (LAML), CCDC6-RET(THCA), SLC45A3-ERG(PRAD), and FGFR3-TACC3(LUSC), consistent with previous reports [67], [68], [69], [70], [71], [72]. Analysis of drug-target fusions revealed 27 drug targets across 29 cancer types present in > 1 % of the samples, with 31 high telomerase activity group specific and 40 low telomerase activity group specific. The *TMPRSS2* and *MET* fusions were significantly enriched in high telomerase activity tumors in PRAD and KIRP, whereas *ROS*, *RET*, *PML-RARA,* and *FGFR3* fusions were enriched in low telomerase activity tumors in LUAD, THCA, LAML, and LUSC (Fisher’s exact test; 95 % CI; *P* < 0.05; Fig. 3b).

### 2.4 Association of telomerase activity with senescence in cancer

Cellular senescence plays a complex role in cancer, exhibiting both tumor-suppressive and tumor-promoting characteristics, and has been proposed as an emerging hallmark of cancer [73], [74], [75], [76]. Although senescent cells display low proliferative potential [77], they can escape senescence arrest and resume proliferation [78] by activating telomere maintenance mechanisms, most commonly through telomerase reactivation [79]. Given the close interplay between telomerase and senescence in tumor biology, we next examined their association across multiple cancer types.

**Fig. 4:**
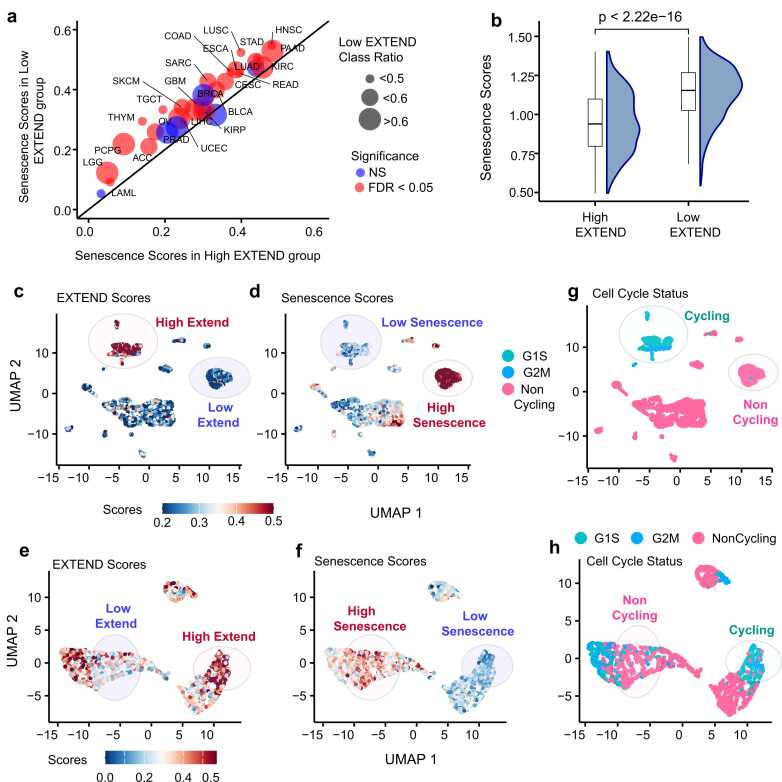
Senescence scores distribution in low and high telomerase activity (EXTEND) groups. (a) Differential senescence score patterns between low (y-axis) and high (x-axis) telomerase activity groups across TCGA pan-cancer data. Circle size represents the proportion of low telomerase activity samples per cancer type. Cancer types with significant differences (FDR < 0.05; Student’s *t*-test) are labeled and shown in red; non-significant (NS) types are shown in blue (b) Differences in senescence scores (y-axis) between low and high telomerase activity groups (x-axis) in cancer cell lines data from the Cancer Cell Line Encyclopedia (CCLE) (*P* < 2.22e−16; Student’s *t*-test). (c-f) UMAP representations of single-cell data showing an inverse relationship between telomerase activity and senescence scores; cells with low telomerase activity (blue) display high senescence scores (red), whereas cells with high telomerase activity (red) show low senescence score (blue). Panels c-d correspond to GBM data (Neftel *et al*., 2019) and panels e-f correspond to HNSC data (Puram *et al*., 2017). (g-h) Cell cycle annotations (cycling vs. non-cycling) for the single-cell GBM (g) and HNSC (h) datasets aligned with the UMAPs in c-f. Representative cell populations are labeled and highlighted in the UMAPs for each case. Source data are provided in the GitHub repository.

We calculated senescence scores (details in methods) for 33 TCGA cancer types using a recently published 125-gene signature [80], comprising of senescence-associated secretory phenotype (SASP) factors and other senescence-related genes. The senescence-associated gene expression signature was significantly enriched in the low telomerase activity group across 26 of 33 cancer types (FDR< 0.05; 95 % CI; Student’s *t*-test; Fig. 4a). Consistent with this trend, telomerase activity showed a significant negative correlation with senescence in pediatric neuroblastoma (Spearman’s *R* = −0.53; *P* = 2.3e-07; Supplementary Fig. 6a) and in cancer cell lines from the Cancer Cell Line Encyclopedia (CCLE; Spearman’s R = −0.57; *P* < 2.2e −16; Supplementary Fig. 6a; Fig. 4b). Across diverse tumor and non-tumor cell lines from CCLE (Supplementary Fig. 6b), fibroblasts exhibited the highest senescence-associated expression and lowest telomerase activity, whereas hematopoietic cell lines displayed the opposite pattern, reflecting their intrinsic self-renewal potential as reported previously [81].

To determine whether these associations also manifest at the single-cell level, we analyzed single-cell RNAseq datasets from glioblastoma (GBM) [82] and head and neck cancers (HNSC) [83]. Using EXTEND derived telomerase activity [29] for these datasets, we observed a strong and significant inverse relationship between senescence and telomerase activity, with higher senescence scores consistently enriched in the low telomerase activity groups (Fig. 4c-d and Fig. 4e-f and Supplementary Fig. 7a and Supplementary 7b; Student’s *t*-test; *P* < 2.2e-16) Analysis of senescence score distributions across cell cycle phases revealed significantly higher senescence activity in noncycling cells in both GBM (Fig. 4g; Supplementary 7c; Student’s *t*-test; *P* < 2.2e-16) and HNSC (Fig. 4h; Supplementary 7d; Student’s *t*-test; *P* < 2.2e-16). These findings confirm that the inverse relationship between senescence and telomerase activity observed at the bulk tumor level is also evident at single-cell resolution, reflecting reduced telomerase activity and increased senescence expression in non-proliferative tumor cell populations.

**Fig. 5:**
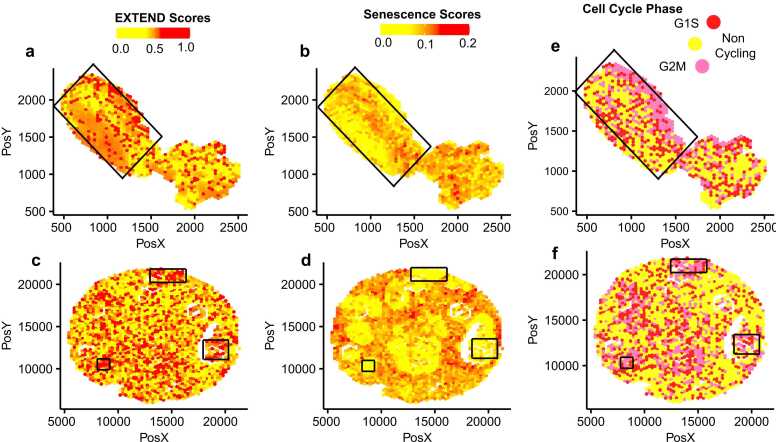
Telomerase activity comparisons in spatial transcriptomics data. Telomerase activity (EXTEND scores) versus senescence scores enrichment across two spatial transcriptomics datasets (a-b) Lung cancer (c-d) Breast Cancer. Cell cycle phase distributions representing cycling and non-cycling cell populations are correlated with senescence and telomerase activity scores for spatial data in (e) Lung cancer and (f) Breast cancer datasets. Black boxes in all panels highlight regions showing enriched patterns of high telomerase activity with low senescence and high cycling activity. Depth of color bar indicates telomerase activity in a and c and senescence scores in b and d. Red and pink colors in e and f represent cycling cell populations and yellow represents non-cycling cells. Source data are provided in the GitHub repository.

To further assess the spatial relationship between telomerase and senescence within tumor tissues, we analyzed spatial transcriptomics datasets from lung [84] and breast [85] tissues. We calculated telomerase, senescence, and cell cycle activity scores for both datasets and compared their spatial enrichment patterns. These analyses revealed opposing spatial distributions of telomerase and senescence activity (Figs. 5a-5d) consistent with the inverse correlation observed in bulk and single-cell transcriptomic datasets. Similarly, cell cycle activity mirrored telomerase activity and was anticorrelated with senescence across spatial regions of both lung (Fig. 5e) and breast tissues (Fig. 5f).

Together, these results demonstrate that telomerase activation and senescence are inversely regulated across cancers, from bulk to single-cell and spatial levels. High telomerase activity or its reactivation appears to suppress senescence programs, consistent with the necessity for tumor cells to bypass senescence to maintain telomere length and sustain proliferation [86]. Although this relationship has been proposed previously [73], our study provided the first quantitative delineation of telomerase-senescence interplay across multiple tumor contexts, including its manifestation at single-cell and spatial resolutions.

### 2.5 Senescence is inversely associated with age in the context of telomerase activity

**Fig. 6:**
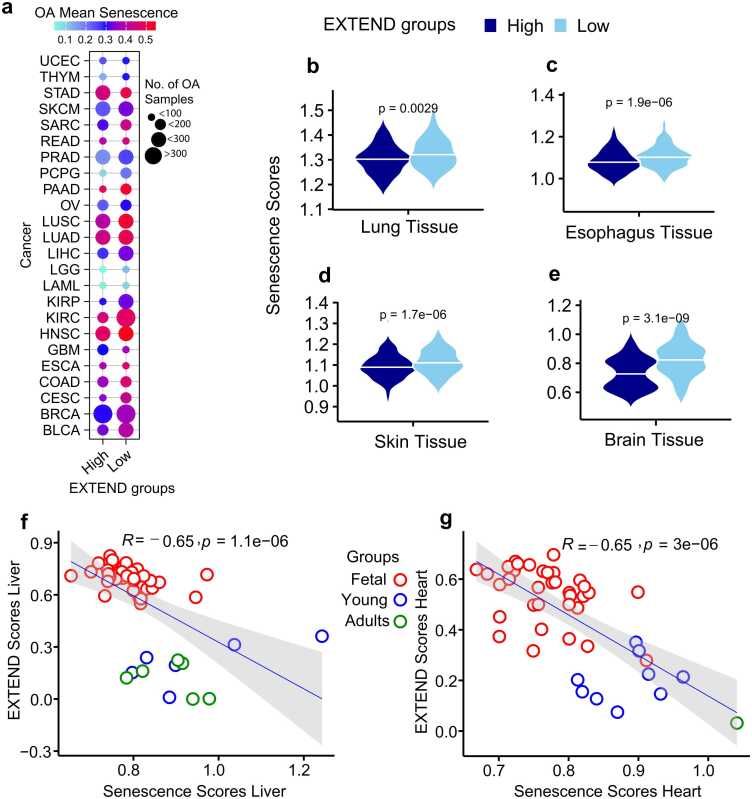
Age-related disparities in senescence and telomerase activity scores. (a) Differential senescence patterns in older adults (OA) stratified by low and high telomerase activity (EXTEND; x-axis) groups across 24 TCGA cancer types (y-axis). The size of each data point represents the number of OA samples in low- or high telomerase activity group, and the color indicates the mean senescence score for OA samples within each group. Only cancer types with significant differences (FDR < 0.05; Student’s *t*-test) are shown. (b-e) GTEx tissues showing senescence score (y-axes) differences in telomerase activity groups (x-axes): (b) lung, (c) esophagus, (d) skin, and (e) brain. *P* values were calculated using Student’s *t*-test. (f-g) Spearman rank correlations between senescence (x-axes) and telomerase activity (y-axes) scores during human development for (f) liver and (g) heart. Colors indicate age groups (red=fetal, blue= young, and green=older adults). Source data are available in the GitHub repository.

To investigate the relationship between cellular senescence and age in the context of telomerase activity, we analyzed cancer types with sufficient sample sizes (n > 50) from TCGA, encompassing 30 cancer types in which older adult (OA) cases could be clearly identified. Comparison of senescence scores between low and high telomerase activity groups in OA patients revealed significant differences across 24 cancer types (Student’s *t*-test, FDR < 0.05; 95 % CI; Fig. 6a). These results are consistent with prior reports suggesting that senescence bypass and telomerase reactivation occur preferentially in tumors with elevated telomerase activity, largely due to increased *TERT* expression driven by promoter mutations or methylation events [87].

To determine whether similar relationships exist in normal tissues, we examined senescence levels using corresponding data from the Genotype Tissue Expression (GTEx) portal. Among tissues with adequate sample sizes in OA individuals, four (lung, esophagus, skin, and brain) exhibited significant differences in senescence between low and high telomerase activity groups (Student’s *t*-test, *P* < 0.001; 95 % CI; Fig. 6b-e). These findings indicate that the interplay between telomerase activity and senescence observed in tumor tissues is mirrored in normal tissues.

We next explored the association between telomerase activity and senescence in human liver and heart tissues, which differ in their intrinsic regenerative capacities during embryonic development [88]. Senescence scores were computed for each tissue and correlated with telomerase activity. Despite limited sample sizes at individual developmental stages, both tissues exhibited strong negative correlations between telomerase activity and senescence (Spearman’s *R* = - 0.65; *P* < 0.0001; 95 % CI; Fig. 6f-g). The significant high telomerase and low senescence in fetal samples in both heart and liver tissue indicate active cell cycle and stemness behavior. Fetal liver and heart tissues displayed high telomerase activity and low senescence scores, consistent with active cell cycling and stem-like behavior. In contrast, this pattern was reversed in younger and older adults, reflecting reduced telomerase activity and elevated senescence, indicative of diminished proliferative capacity with age [89]. Notably, liver tissue exhibited a mixed pattern across adult stages, potentially reflecting its sustained self-renewal potential and residual telomerase activity during these periods [90], [91].

### 2.6 Immune inflammation in cancer is associated with reduced telomerase activity

Telomerase activation supports cellular proliferation and stemness across multiple tumor types [29],it has also been implicated in modulating immune responses and preventing immune cell senescence [92]. To investigate the interplay between telomerase activity and immune dynamics, we analyzed telomerase levels in relation to tumor immune subtypes across TCGA cancer types.

**Fig. 7:**
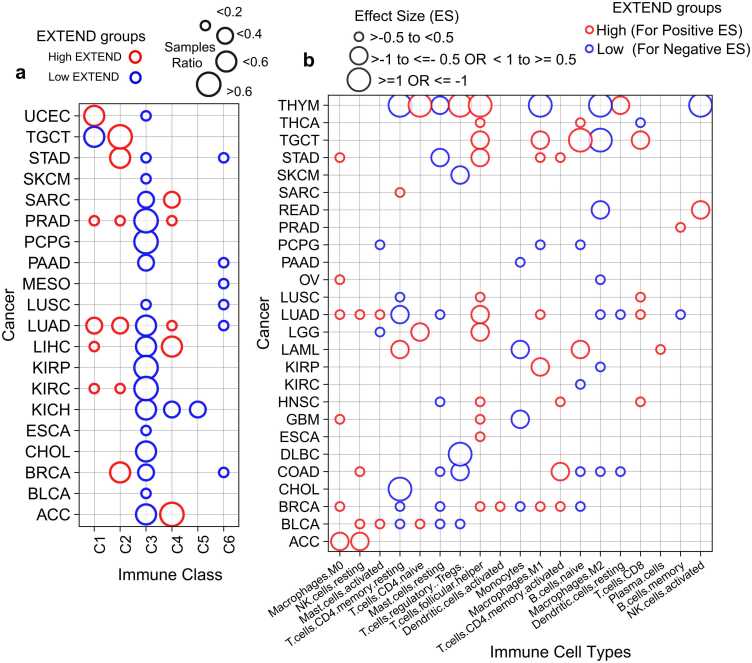
Association of telomerase activity with Immune system. (a) Differential fractions of immune classes (x-axis) between low and high telomerase activity (EXTEND) groups across 20 TCGA cancer types (y-axis). Circle size represents the proportion of samples in each telomerase activity group; red indicates high and blue indicates low telomerase activity group. Only significant cases (FDR < 0.05; Fisher’s exact test) are shown. (b) Differences in immune cell type (x-axis) abundances between low and high telomerase activity groups across 26 cancer types (y-axis).Circle size represents effect size (change between low and high telomerase activity values). Blue color indicates negative effect sizes (associated with low telomerase activity), and red color indicate positive effect sizes (associated with high telomerase activity). Only Significant cases (FDR <0.05; Student’s *t*-test) are shown. Source data are provided in the GitHub repository.

Immune subtypes were obtained from a previously published pan-cancer classification [93], defined by features including immune gene expression signatures, macrophage and lymphocyte infiltration, proliferation indices, and intratumoral heterogeneity. Across 18 cancer types, tumors with low telomerase activity were significantly enriched for the C3 immune subtype (Fisher’s exact test, FDR < 0.05; 95 % CI; Fig. 7a) which represents the low-proliferative, inflammatory immune subtype. Conversely, a smaller subset of cancers exhibited enrichment for the high-proliferative immune classes C1 and C2. Testicular germ cell tumors (TGCT) were an exception, with both high (72 %) and low (28 %) telomerase activity groups mapping to highly proliferative immune classes, consistent with the overall proliferative nature of testicular tissue. The lymphocyte-depleted C4 subtype was associated with high telomerase activity in sarcoma (SARC), adrenocortical carcinoma (ACC), and three additional cancer types, while the immunologically quiet subtype (C5) was observed primarily in kidney chromophobe (KICH). A small fraction of tumors with low telomerase activity from liver, lung, mesothelioma, pancreas, stomach, and breast cancers were classified as TGF-β–dominant (C6), consistent with immunosuppressive tumor microenvironments.

To further delineate immune-telomerase associations at the cellular level, we used immune cell-type deconvolution scores derived from TCGA pan-cancer data using CIBERSORT [94]. Activated T-cells were predominantly associated with high telomerase activity across most cancer types, whereas resting T-cells were enriched in tumors with low telomerase activity, corroborating previous observations [95], [96]. Similarly, macrophage polarization was linked to telomerase levels: M0 and M1 macrophages were enriched in high telomerase activity tumors (Fig. 7b; Supplementary Figs.8a-b), while M2 macrophages and resting mast cells are preferentially associated with low telomerase activity across seven cancer types (Fig. 7b and Supplementary Fig.9a). Monocytes also exhibited low telomerase activity (Student’s *t*-test; FDR < 0.05; 95 % CI; Fig. 7b; Supplementary Fig. 9b), consistent with previous reports linking monocytes to senescence phenotypes [80], [97]. To validate these observations at single-cell resolution, we analyzed immune populations across ten cancer types. Proliferating immune cells displayed significantly higher telomerase activity than non-proliferating immune cells (Student’s *t*-test P < 2.2e-16; 95 % CI; Supplementary Fig.10), confirming the bulk level associations.

**Fig. 8:**
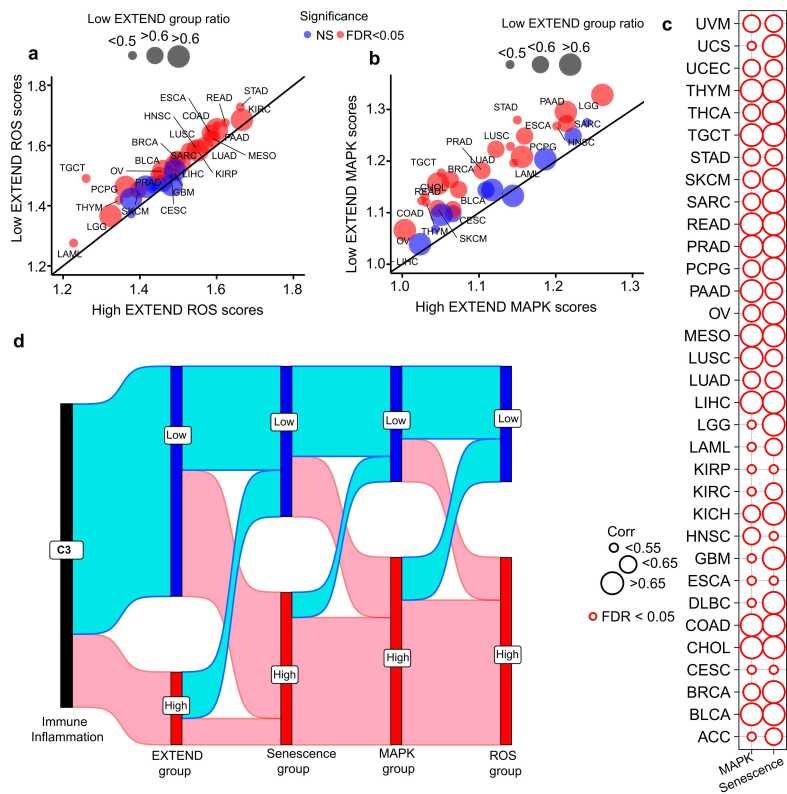
Summary of telomerase activity associations. (a) Differential patterns of low (y-axes) and high (x-axes) telomerase activity (EXTEND) groups across TCGA pan-cancer for (a) ROS scores and (b) MAPK signaling scores. Cancer types with significant differences (FDR < 0.05; Student’s *t*-test) are highlighted in red; non-significant (NS) cases are shown in blue. Circle size represents the fraction of samples in low telomerase activity group for each cancer type. (c) Spearman correlations of ROS scores with MAPK and senescence scores across 33 TCGA cancer types. Circle size represents the magnitude of correlation coefficient; all correlations are significant after FDR correction (FDR < 0.05) (d) Sankey plot representing the association summary of immune class C3 with low and high telomerase, senescence, MAPK, and ROS activity groups across TCGA pan-cancer. Source data are provided in the GitHub repository.

Emerging evidence indicates that mitochondrial dysfunction and reactive oxygen species (ROS) are the key drivers of senescence phenotype [98], [99], primarily through activation of the MAPK signaling cascade [100], [101], [102]. Consistent with this, our pathway enrichment analysis revealed MAPK signaling as one of the top pathways associated with low telomerase activity group (Supplementary Fig.4). Given the connectivity of the mentioned pathways, we next examined enrichment of ROS and MAPK signaling between low and high telomerase activity groups across TCGA cancers. Using gene sets from MSigDB [64] for these pathways, we calculated their gene set enrichment analysis scores. The differential analysis of both ROS and MAPK scores exhibited significant enrichment for low telomerase activity groups across 33 cancer types (Student’s *t*-test, FDR < 0.05; 95 % CI; Fig. 8a-b).

We next examined the association of ROS and senescence across TCGA cancers. We noted that, at a pan-cancer level ROS and senescence were strongly correlated (*Rho* = 0.9, *P* = 9.94 E-09), a relationship that persisted within individual tumor types (Spearman’s FDR < 0.05; Fig. 8c), suggesting a lineage-independent mechanism. A similar trend was observed between ROS and MAPK signaling (Spearman’s FDR <0.05; Fig. 8c). These associations highlight the links between telomerase activity, senescence, MAPK, and ROS mechanisms. Notably, when connected with immune inflammation, we identified that the C3 immune subtype, characterized by low telomerase activity (Fig. 7a), exhibited the highest levels of senescence, ROS accumulation, and MAPK pathway activation (Fig. 8d). Together, these findings indicate that tumors with low telomerase activity are defined by an inflammatory, ROS-enriched microenvironment and enhanced senescence signaling, establishing a functional link between telomerase repression, immune activation, and cellular aging across diverse cancer lineages (Fig. 8d).

## 3 Discussion

Telomerase, a central regulator of multiple cancer hallmarks [19], [103], [104], [105], [106], is active in highly proliferative cells such as germ and stem cells, as well as in approximately 90 % of all human cancers [107], [108]. Some normal cells also exhibit detectable levels of telomerase activity, which in certain cases is associated with the proliferative capacity of specific cell populations in tissues that require a high cell turnover [109]. It is widely accepted that sustained tumor proliferation depends on the maintenance of telomere stability [34], [62], [103], [108]. Hence, most cancers bypass cellular senescence by activating telomerase or employing alternative lengthening of telomeres (ALT) mechanisms to preserve telomere length [110]. These insights underscore the significance of telomerase not only as a diagnostic biomarker but also as a promising target for anticancer therapies.

Although telomerase has been extensively studied across various malignancies [19], [103], [104], [105], [106], its heterogeneous expression across tissues and the distinct regulatory mechanisms influencing its activity remain underexplored, creating a notable knowledge gap. By quantifying telomerase activity using EXTEND [29], we established a robust framework for stratifying tumors into low and high telomerase activity groups across a broad spectrum of cancers.

In this study, using an unsupervised data-driven approach, we classified tumors into low and high telomerase activity groups across a comprehensive collection of cohorts, integrating bulk, single-cell and spatial transcriptomics datasets. We then systematically compared their clinical and genomic characteristics across multiple cancer types. We did not observe substantial differences between these groups in clinical variables such as sex, age, and disease stage, with a few exceptions. In contrast, tumors with high telomerase activity generally exhibited greater genomic instability, including higher tumor mutation burden, increased loss of heterozygosity and more frequent copy number alterations. Thymoma was an exception to the trend. These patterns are consistent with previous studies that used *TERT* expression or *TERT* promoter mutations as surrogate for high telomerase activity [48], [111].

In our previous analysis [29], telomere length showed no significant association with telomerase activity. Here, by re-examining the same datasets using a different approach - stratifying tumors into low and high telomerase activity groups - we observed a significant enrichment of low telomerase activity with longer telomere lengths, again with thymoma as an outlier. This pattern reinforces the inverse relationship between telomere length and telomerase activation as reported previously [112] and highlights the added interpretive power of our categorical framework.

Classification of bulk tumors and single cells datasets into low and high telomerase activity groups confirmed previously observed trends, and revealed genomic gradients not systematically characterized at a pan-cancer level [48], [111], [113]. As shown in prior studies, [47], [63] *CIC* mutations are enriched in *TERT* promoter mutant low-grade gliomas, whereas *ATRX* mutations occur in *TERT* wild type, a pattern consistent with our high and low telomerase activity groups across cancers.

A striking feature of our analysis was the enrichment of senescence, a marker of cell cycle arrest, in tumors with low telomerase activity, reflecting their reduced proliferative potential. This association was corroborated in cancer cell lines, and across single-cell and spatial transcriptomics analyses. These findings align with a prior pan-cancer study showing that senescence is anticorrelated with genomic instability and copy number alterations [97], which were highly enriched in our high telomerase activity groups. Beyond its canonical role in telomere maintenance, telomerase exerts non-canonical effects, including suppression of reactive oxygen species (ROS) and prevention of senescence phenotype [114], [115]. Elevated telomerase levels have been shown to inhibit senescence and apoptosis in human endothelial cells [116], [117], whereas telomerase deficiency increases ROS generation and oxidative damage [114]. These findings are consistent with inverse association of senescence with telomerase activity in our analysis framework.

We observed a significant association between low telomerase activity and elevated senescence in older adults, across both cancerous and normal tissues, highlighting the impact of cellular aging on telomerase regulation as observed by previous studies [73], [118], [119]. The link between immune inflammation and the low telomerase activity group across majority of cancers indicates its association with ROS generation and related mechanisms as highlighted in an earlier study [120]. Moreover, the robust association of ROS and MAPK signaling with senescence across 33 cancers in our study corroborates reported studies [121], [122], [123], [124], [125].These strong associations suggest coordinated regulation of these processes in senescent, low telomerase activity tumors.

From a clinical standpoint, telomerase activity and senescence appear to play a dual and context-dependent role in tumor biology. While reduced telomerase activity may limit cellular proliferation and tumor growth through induction of senescence, the accumulation of senescent cells and the accompanying senescence-associated secretory phenotype (SASP) can promote a pro-inflammatory and immunosuppressive tumor microenvironment. Such changes may enhance tumor heterogeneity, facilitate immune evasion, and influence resistance to therapy. Conversely, cancers that maintain high telomerase activity often exhibit sustained proliferative potential but may remain more susceptible to telomerase-targeted or DNA-damaging therapies. Therefore, integrating telomerase activity and senescence status into clinical stratification frameworks could improve prognostic precision and guide the design of therapeutic interventions aimed at modulating cellular aging, immune responses, and treatment sensitivity in cancer [73], [74], [75], [76], [86].

A limitation of our study is the lack of direct comparison between telomerase activity groups and alternative lengthening of telomeres (ALT) mechanisms. This omission reflects the current absence of a comprehensive, pan-cancer ALT classification or a validated expression-based ALT signature. Evidence suggests that ALT-positive tumors or tumors lacking any active telomere maintenance mechanism, such as those observed in sarcomas and neuroblastomas [20], [126], may also occur across other malignancies. However, the systematic identification of these tumors requires validated expression-based markers and robust tumor annotations. As improved ALT classifiers and molecular signatures become available, future analyses integrating both telomerase-dependent and ALT-mediated telomere maintenance mechanisms will be critical to fully elucidate the landscape of telomere regulation across cancers.

In summary, our study provides a pan-cancer framework for integrating genomic, transcriptomic, and cellular features to define distinct molecular states of telomerase activity. By linking telomerase activity groups with genomic instability, cellular senescence, and survival outcomes, we offer deeper insights into the regulation of telomerase across diverse malignancies [127]. Collectively, these findings position telomerase activity as a central determinant of tumor behavior and a clinically actionable axis with significant potential for therapeutic targeting.

## 4 Methods

### 4.1 Data sources

Bulk RNA sequencing (RNA-seq) data and associated phenotypic information for 33 cancer types were obtained from The Cancer Genome Atlas (TCGA; Synapse ID: syn4874822; https://gdc.cancer.gov/node/905/). The included cancer types were ACC, BLCA, BRCA, CESC, CHOL, COAD, DLBC, GBM, HNSC, ESCA, KICH, KIRC, KIRP, LAML, LGG, LIHC, LUAD, LUSC, MESO, OV, PCPG, PRAD, PAAD, SARC, SKCM, STAD, READ, TGCT, THYM, THCA, UCEC, UVM, and UCS. Details of cancer types abbreviations are mentioned in the results section.

Gene expression data for cancer cell lines were retrieved from the Cancer Cell Line Encyclopedia (CCLE; https://portals.broadinstitute.org/ccle, version released 02-Jan-2019). Normal tissue RNA-seq data were obtained from Genotype Tissue Expression (GTEx) project (release v7, downloaded March 2019). Bulk RNA-seq data for human developmental liver and heart tissues were downloaded from ArrayExpress (accession no: E-MTAB-6814) [88].

Single-cell RNA-seq datasets for glioblastoma (GBM) and head and neck cancers (HNSC) were obtained from previously published studies [82], [83]. Spatial transcriptomics data for lung and breast cancers were retrieved from prior studies [84], [85]. Additionally, single-cell immune transcriptomic profiles covering ten cancer types were obtained from a previously published dataset [128].

No human subjects were directly involved in this study; all analyses were conducted on publicly available data.

### 4.2 Unsupervised classification of telomerase activity scores

Telomerase activity was quantified using EXTEND scores as described previously [29] for all bulk, single-cell, and spatial transcriptomic datasets. Briefly, the EXTEND algorithm [29] uses gene expression matrices as input to estimate telomerase activity. The result is generated in the form of a table representing sample-based telomerase activity scores. The method is implemented as an R package available at the GitHub repository (https://github.com/NNoureen/EXTEND). The details of EXTEND method, installation instructions and it’s limitations are mentioned in the previous study [29].

Telomerase activity scores (EXTEND scores) were stratified into two groups representing low and high telomerase activity groups using an unsupervised *k*-means [50] clustering approach implemented in R. Clustering was performed independently for each cancer type or dataset, with the number of clusters fixed at *k* = 2, representing low and high telomerase activity groups. Input to the *k*-means function was a linear vector of telomerase activity scores. We used *k*-means clustering, as compared to fixed-threshold methods (e.g., median splits), it provides data-driven classification that accommodates non-uniform score distributions.

To ensure robustness, the clustering algorithm was executed for 1000 iterations, each with 50 random centroid initializations, resulting in 50,000 total iterations per dataset. Final classification of samples into “low” and “high” telomerase activity groups was determined based on consensus across all runs.

### 4.3 Clinical and demographic variables comparisons

We examined associations between telomerase activity and key clinical and demographic variables across TCGA cancer types. Variables include patient age, sex, tumor stage, and overall survival. All analyses compared samples stratified into low and high telomerase activity groups, as defined by unsupervised *k*-means clustering of EXTEND scores.

Samples were categorized as young adults (YA; 18–50 years) or older adults (OA; >50 years) for age-based comparisons. Cancer types with fewer than 15 cases, missing age data, or predominantly pediatric cohorts were excluded, resulting in 28 cancer types for comparison. Associations between age group and telomerase activity were assessed using Fisher’s exact test.

Sex-based comparisons were performed across cancer types after excluding gender-specific malignancies (e.g., BRCA, PRAD, OV, and UCEC). This resulted in 26 cancer types for analysis. Associations between sex (male vs. female) and telomerase activity groups were evaluated using Fisher’s exact test.

Tumor stage data were harmonized by combining stages I-II into “low-stage” and stages III-IV into “high-stage” to ensure adequate group sizes. Twenty-one cancer types with available staging information were included in the analyses. Fisher’s exact test was used to assess associations between telomerase activity groups and tumor stage.

Overall survival analyses were performed for all 33 TCGA cancer types using a Cox proportional hazards regression model implemented in R. Hazard ratios were estimated for low versus high telomerase activity groups and significance was evaluated using the log-rank test. Unlike our previous analysis [29], which employed a median-based stratification across 31 cancer types, the current analysis used the unsupervised classification of telomerase activities across all 33 cancers.

### 4.4 Genome instability comparisons

Genome instability was compared between low and high telomerase activity groups across 33 TCGA cancer types. The genomic features analyzed include telomere length (TL), tumor mutation burden (TMB), copy number altered fraction (CNA), loss of heterozygosity (LOH), whole-genome doubling (WGD), and gene fusion events.

Telomere length estimates were obtained from a previous study [47] and represent the genome-wide average TL per sample, calculated as the mean across all chromosome ends. To assess relative telomere shortening or elongation, we used tumor-to-normal ratio (TL ratio) as a comparative metric, available for 32 TCGA cancer types.

Data for TMB, CNAs, LOH, and WGD were obtained from the TCGA Pan-Cancer Immune Landscape resource (https://gdc.cancer.gov/about-data/publications/panimmune). Information on gene fusions and druggable fusion events was retrieved from the TCGA fusion resource study [66]. Comparisons of genomic features between low and high telomerase activity groups were performed using Fisher’s exact test for binary and categorical variables (e.g., presence of absence of gene fusions, WGD, recurrent fusions, or druggable fusion events) and two-sided *t*-tests for continuous variables (e.g., TL, CNA, TMB, and LOH). Multiple testing correction was applied using the False Discovery Rate (FDR) method.

To further explore TMB patterns, we analyzed cancer types with significant TMB differences between low and high telomerase activity groups using the TCGA mutation file “mc3.v0.2.8.PUBLIC.maf”. Using maftools (v2.18.0), we identified the top 20 frequently mutated genes for each cancer type and scrutinized them for recurrent (genes mutated in two or more cancer types) and non-recurrent cancer-specific mutations. This analysis identified 16 recurrently mutated genes across 17 cancer types and 28 cancer-specific mutations spanning 11 cancer types. Subsequently, we compared the frequencies of recurrent and non-recurrent cancer-specific gene mutations between low and high telomerase activity groups across the corresponding TCGA cancer types.

Frequencies of *TERT* promoter mutations and *ATRX* gene mutations were compared between telomerase activity groups across TCGA pan-cancer data using data from a previous study [47]. In low grade glioma (LGG), ATRX-mutant and TERT-promoter mutant samples were further compared for genome instability features and telomerase activity scores using two-sided *t*-tests.

Finally, to assess pathway-level differences, differential gene expression analysis was performed using edgeR [129] between low and high telomerase activity groups across TCGA pan-cancer. The top 100 upregulated genes from each group were subjected to pathway enrichment analysis using MSigDB.

### 4.5 Senescence score calculation and comparison

Senescence scores were computed using the SenMayo gene set comprising 125 senescence- associated genes from a recent study [80].For bulk RNA-seq data sets (TCGA, GTEx, CCLE, and human developmental data), senescence scores were calculated using single-sample Gene Set Enrichment Analysis (ssGSEA) implemented in the GSVA [130] R package. For single-cell GBM and HNSC datasets, senescence scores were derived using the JASMINE [131] single-cell signature scoring method following the standard protocol [132]. For spatial transcriptomics datasets from lung and breast cancer, senescence scores were computed using the module based scoring method AUCell [133].

Single-cell and spatial transcriptomics data were processed with Seurat [134] using default procedures for normalization, scaling, and cell cycle scoring. Differences in senescence scores between low and high telomerase activity groups were assessed with two-sided *t*-tests and *P*-values were adjusted for multiple testing using the FDR method. Correlations between telomerase activity and senescence scores were assessed using Spearman’s rank correlation. Uniform Manifold Approximation and Projection (UMAP) features implemented in Seurat were used to visualize and compare telomerase activity with senescence and cell cycle features in both single-cell and spatial transcriptomics data sets.

To explore age-related patterns, samples in TCGA and GTEx were categorized as younger adults (adolescents and young adults, AYA; 15–39 years) and older adults (OA; >39 years) according to a recent classification [135]. Due to limited AYA representation and its sparse distribution across 33 cancer types and two telomerase activity groups, only OA samples with more than 50 cases per cancer type were included, resulting in 30 TCGA cancer types. For GTEx data, TCGA cancer types with matched normal tissues and sufficient samples sizes (> 50 samples per telomerase activity group) were included in the age-related comparison, yielding four tissues (brain, esophagus, lung, and skin).

Comparisons of senescence scores between low and high telomerase activity groups were conducted using two-sided *t*-tests. Correlations between senescence and telomerase activity were assessed using Spearman’s method for human developmental dataset.

### 4.6 Immune cell types and their association with telomerase activity groups

Immune classes were defined based on the TCGA immunogenomic classification [93], which categorized tumor samples into six immune subtypes: C1 (wound healing), C2 (INF-ˠ dominant), C3 (Inflammatory), C4(Lymphocyte depleted), C5(Immunologically quite), and C6(TGF-β dominant). Associations between low and high telomerase activity groups and immune subtype frequency distributions were evaluated across 30 available cancer types using Fisher’s exact test. In addition, immune cell abundance estimates for 33 TCGA cancer types were obtained using the ESTIMATE algorithm. Differences in immune cell proportions between telomerase activity groups were evaluated using two-sided t-tests. All *P*-values were corrected for multiple testing using FDR.

To further investigate whether single-cell data reflected similar trends observed in bulk tissue, we analyzed telomerase activity scores in immune cells derived from tumor microenvironment across 10 cancer types using single-cell RNAseq datasets. Proliferating immune cells were compared to non-proliferating immune cells within each cancer type using two-sided *t*-tests. Immune cells characterization and corresponding single-cell data were obtained from a recently published study [128].

### 4.7 MAPK and reactive oxygen species (ROS) scores association with telomerase and senescence

Gene sets representing the MAPK signaling and Reactive Oxygen Species (ROS) pathways were obtained from the molecular signatures database (MSigDB). Single-sample gene set Enrichment Analysis (ssGSEA) [130] was performed to compute pathway activity scores across 33 TCGA cancer types.

The resulting MAPK and ROS scores were compared with senescence scores across TCGA samples using Spearman’s rank correlation to evaluate their association Subsequently, differences in MAPK and ROS pathway activities between low and high telomerase activity groups were assessed for each cancer type using two-sided *t*-tests.

To further investigate the interplay between immune context, telomerase activity, and stress signaling, we examined the C3(inflammatory) immune subtype, which was the most prevalent within low telomerase activity group across TCGA pan-cancer data. We evaluated the distribution and relationship of telomerase activity, senescence, MAPK signaling, and ROS activation within this immune context across TCGA cancers. For visualization, MAPK, ROS, and senescence scores were stratified into low and high groups using *k*-means clustering, consistent with the approach applied for telomerase activity scoring. The interconnections among these pathways across TCGA cancer types were visualized using Sankey plot generated in R.

## Data and materials availability

Datasets used in this project are from publicly available resources and are mentioned in the Data sources in the methods section. Data and scripts used to generate the main and supplementary figures are provided in the GitHub repository https://github.com/NNoureen/Telomerase-Stratification. Some data files are provided in the zenodo repository due to file size limitations https://doi.org/10.5281/zenodo.17556000.

## Author contributions

N.N. and M.H.K. conceptualized and designed the research; N.N collected and analyzed the data; N.N. and M.H.K. wrote the manuscript.

## CRediT authorship contribution statement

**Min Hee Kang:** Writing – review & editing, Conceptualization. **Nighat Noureen:** Writing – review & editing, Writing – original draft, Visualization, Validation, Supervision, Software, Resources, Project administration, Methodology, Investigation, Funding acquisition, Formal analysis, Data curation, Conceptualization.

## Ethical approval

This declaration is not applicable as no experimental subjects were involved in the study.

## Funding

The work was funded by the 10.13039/100004917Cancer Prevention and Research Institute of Texas (TREC RP210154 to C. Patrick Reynolds, and N.N is the project lead and M.K. is the CO-PI).

## Declaration of Competing Interest

The authors declare no competing interests.

## References

[1] Blackburn E.H. (1991). Structure and function of telomeres. Nature.

[2] Levy M.Z., Allsopp R.C., Futcher A.B., Greider C.W., Harley C.B. (1992). Telomere end-replication problem and cell aging. J Mol Biol.

[3] Lazzerini-Denchi E., Sfeir A. (2016). Stop pulling my strings—what telomeres taught us about the DNA damage response. Nat Rev Mol Cell Biol.

[4] Sahin E., DePinho R.A. (2010). Linking functional decline of telomeres, mitochondria and stem cells during ageing. Nature.

[5] Greider C.W. (1998). Telomeres and senescence: the history, the experiment, the future. Curr Biol.

[6] Artandi S.E., DePinho R.A. (2010). Telomeres and telomerase in cancer. Carcinogenesis.

[7] Hanahan D., Weinberg R.A. (2011). Hallmarks of cancer: the next generation. Cell.

[8] Greider C.W. (1991). Telomerase is processive. Mol Cell Biol.

[9] Broccoli D., Young J.W., de Lange T. (1995).

[10] Nugent C.I., Lundblad V. (1998). The telomerase reverse transcriptase: components and regulation. Genes Dev.

[11] Feng J. (1995). The RNA component of human telomerase. Science.

[12] Mitchell J.R., Wood E., Collins K. (1999). A telomerase component is defective in the human disease dyskeratosis congenita. Nature.

[13] Huang F.W. (2013). Highly recurrent promoter mutations in human melanoma. Science.

[14] Killela P.J. (2013). promoter mutations occur frequently in gliomas and a subset of tumors derived from cells with low rates of self-renewal. P Natl Acad Sci.

[15] Akincilar S.C. (2016). Long-range chromatin interactions drive mutant promoter activation. Cancer Discov.

[16] Huang F.W. (2015). Promoter mutations and monoallelic activation of in cancer. Oncogenesis.

[17] Stern J.L. (2017). Allele-specific DNA methylation and its interplay with repressive histone marks at promoter-mutant Genes. Cell Rep.

[18] Stern J.L., Theodorescu D., Vogelstein B., Papadopoulos N., Cech T.R. (2015). Mutation of the promoter, switch to active chromatin, and monoallelic expression in multiple cancers. Gene Dev.

[19] Borah S. (2015). TERT promoter mutations and telomerase reactivation in urothelial cancer. Science.

[20] Koneru B. (2020). Telomere maintenance mechanisms define clinical outcome in high-risk neuroblastoma. Cancer Res.

[21] Avilion A.A. (1996). Human telomerase RNA and telomerase activity in immortal cell lines and tumor tissues. Cancer Res.

[22] Bell R.J.A. (2015). The transcription factor GABP selectively binds and activates the mutant TERT promoter in cancer. Science.

[23] Horn S. (2013). Promoter mutations in familial and sporadic melanoma. Science.

[24] Vinagre J. (2013). Frequency of promoter mutations in human cancers. Nat Commun.

[25] Hrdlicková R., Nehyba J., Bose H.R. (2012). Alternatively spliced telomerase reverse transcriptase variants lacking telomerase activity stimulate cell proliferation. Mol Cell Biol.

[26] Liu K., Hodes R.J., Weng N.-p (2001). Cutting edge: telomerase activation in human T lymphocytes does not require increase in telomerase reverse transcriptase (hTERT) protein but is associated with hTERT phosphorylation and nuclear translocation. J Immunol.

[27] Ludlow A.T., Slusher A.L., Sayed M.E. (2019). Insights into telomerase/hTERT alternative splicing regulation using bioinformatics and network analysis in cancer. Cancers.

[28] Ludlow A.T. (2018). NOVA1 regulates splicing and cell growth in non-small cell lung cancer. Nat Commun.

[29] Noureen N. (2021). Integrated analysis of telomerase enzymatic activity unravels an association with cancer stemness and proliferation. Nat Commun.

[30] Rowland T.J., Dumbovic G., Hass E.P., Rinn J.L., Cech T.R. (2019). Single-cell imaging reveals unexpected heterogeneity of telomerase reverse transcriptase expression across human cancer cell lines. P Natl Acad Sci.

[31] Ulaner G.A., Hu J.F., Vu T.H., Giudice L.C., Hoffman A.R. (1998). Telomerase activity in human development is regulated by human telomerase reverse transcriptase (hTERT) transcription and by alternate splicing of hTERT transcripts. Cancer Res.

[32] Xi L.H., Cech T.R. (2014). Inventory of telomerase components in human cells reveals multiple subpopulations of hTR and hTERT. Nucleic Acids Res.

[33] Ramirez R.D., Wright W.E., Shay J.W., Taylor R.S. (1997). Telomerase activity concentrates in the mitotically active segments of human hair follicles. J Invest Dermatol.

[34] Kim N.W. (1994). Specific association of human telomerase activity with immortal cells and cancer. Science.

[35] Cherif H., Tarry J.L., Ozanne S.E., Hales C.N. (2003). Ageing and telomeres: a study into organ- and gender-specific telomere shortening. Nucleic Acids Res.

[36] Demanelis K. (2020). Determinants of telomere length across human tissues. Science.

[37] Kesäniemi J. (2019). Exposure to environmental radionuclides associates with tissue-specific impacts on telomerase expression and telomere length. Sci RepUk.

[38] Seluanov A. (2007). Telomerase activity coevolves with body mass not lifespan. Aging Cell.

[39] Albanell J. (1997). High telomerase activity in primary lung cancers: association with increased cell proliferation rates and advanced pathologic stage. J Natl Cancer Inst.

[40] Yoshida K. i, Sakamoto S. i, Sumi S., Higashi Y., Kitahara S. (1998). Telomerase activity in renal cell carcinoma. Cancer Interdisciplinary Int J Am Cancer Soc.

[41] Saretzki G. (2014). Extra-telomeric functions of human telomerase: cancer, mitochondria and oxidative stress. Curr Pharm Des.

[42] Ghosh A. (2012). Telomerase directly regulates NF-κB-dependent transcription. Nat Cell Biol.

[43] Park J.-I. (2009). Telomerase modulates Wnt signalling by association with target gene chromatin. Nature.

[44] Ghareghomi S., Ahmadian S., Zarghami N., Kahroba H. (2021). Fundamental insights into the interaction between telomerase/TERT and intracellular signaling pathways. Biochimie.

[45] Palamarchuk A.I., Kovalenko E.I., Streltsova M.A. (2023). Multiple actions of telomerase reverse transcriptase in cell death regulation. Biomedicines.

[46] Smogorzewska A., de Lange T. (2002). Different telomere damage signaling pathways in human and mouse cells. Embo J.

[47] Barthel F.P. (2017). Systematic analysis of telomere length and somatic alterations in 31 cancer types. Nat Genet.

[48] Luo Z. (2019). Pan-cancer analysis identifies telomerase-associated signatures and cancer subtypes. Mol Cancer.

[49] Sieverling L. (2020). Genomic footprints of activated telomere maintenance mechanisms in cancer. Nat Commun.

[50] Likas A., Vlassis N., Verbeek J.J. (2003). The global k-means clustering algorithm. Pattern Recognit.

[51] Amini M., Sharma R., Jani C. (2023). Gender differences in leukemia outcomes based on health care expenditures using estimates from the GLOBOCAN 2020. Arch Public Health.

[52] Bellenghi M. (2020). Sex and gender disparities in melanoma. Cancers.

[53] May L., Shows K., Nana-Sinkam P., Li H.W., Landry J.W. (2023). Sex differences in lung cancer. Cancers.

[54] Park J.O. (2022). Sex differences in the prevalence of head and neck cancers: a 10-year follow-up study of 10 million healthy people. Cancers.

[55] Jeon H.S. (2012). Telomerase activity and the risk of lung cancer. J Korean Med Sci.

[56] Kim S. (2022). The telomere maintenance mechanism spectrum and its dynamics in gliomas. Genome Med.

[57] Liu T. (2014). The age- and shorter telomere-dependent TERT promoter mutation in follicular thyroid cell-derived carcinomas. Oncogene.

[58] Mekhail T.M. (2003). Renal cell carcinoma (RCC) and telomerase activity: relationship to stage. Urol OncolSemin Ori.

[59] Onoda N. (1998). Telomerase activity in thyroid tumors. Oncol Rep.

[60] Knijnenburg T.A. (2018). Genomic and molecular landscape of DNA damage repair deficiency across the cancer genome atlas. Cell Rep.

[61] Zheng S.Y. (2016). Comprehensive pan-genomic characterization of adrenocortical carcinoma (vol 29, pg 723, 2016). Cancer Cell.

[62] López-Otín C., Blasco M.A., Partridge L., Serrano M., Kroemer G. (2013). The hallmarks of aging. Cell.

[63] Brat D.J. (2015). Comprehensive, integrative genomic analysis of diffuse lower-grade gliomas. N Engl J Med.

[64] Liberzon A. (2011). Molecular signatures database (MSigDB) 3.0. Bioinformatics.

[65] Subramanian A. (2005). Gene set enrichment analysis: a knowledge-based approach for interpreting genome-wide expression profiles. P Natl Acad Sci.

[66] Gao Q. (2018). Driver fusions and their implications in the development and treatment of human cancers. Cell Rep.

[67] De Braekeleer E., Douet-Guilbert N., De Braekeleer M. (2014). RARA fusion genes in acute promyelocytic leukemia: a review. Expert Rev Hematol.

[68] Esgueva R. (2010). Prevalence of TMPRSS2–ERG and SLC45A3–ERG gene fusions in a large prostatectomy cohort. Mod Pathol.

[69] Kim P. (2022). FusionGDB 2.0: fusion gene annotation updates aided by deep learning. Nucleic Acids Res.

[70] Liquori A. (2020). Acute promyelocytic leukemia: a constellation of molecular events around a single PML-RARA fusion gene. Cancers.

[71] Santoro M., Moccia M., Federico G., Carlomagno F. (2020). RET gene fusions in malignancies of the thyroid and other tissues. Genes.

[72] Vodopivec D.M., Hu M.I. (2022). RET kinase inhibitors for RET-altered thyroid cancers. Ther Adv Med Oncol.

[73] Campisi J. (2013). Aging, cellular senescence, and cancer. Annu Rev Physiol.

[74] Domen A. (2022). Cellular senescence in cancer: clinical detection and prognostic implications. J Exp Clin Cancer Res.

[75] Pérez-Mancera P.A., Young A.R., Narita M. (2014). Inside and out: the activities of senescence in cancer. Nat Rev Cancer.

[76] Zeng S., Shen W.H., Liu L. (2018). Senescence and cancer. Cancer Transl Med.

[77] Sun L. (2019).

[78] Romanov S.R. (2001). Normal human mammary epithelial cells spontaneously escape senescence and acquire genomic changes. Nature.

[79] Bednarek A., Budunova I., Slaga T.J., Aldaz C.M. (1995). Increased telomerase activity in mouse skin premalignant progression. Cancer Res.

[80] Saul D. (2022). A new gene set identifies senescent cells and predicts senescence-associated pathways across tissues. Nat Commun.

[81] Morrison S.J., Prowse K.R., Ho P., Weissman I.L. (1996). Telomerase activity in hematopoietic cells is associated with self-renewal potential. Immunity.

[82] Neftel C. (2019). An integrative model of cellular states, plasticity, and genetics for glioblastoma. Cell.

[83] Puram S.V. (2017). Single-cell transcriptomic analysis of primary and metastatic tumor ecosystems in head and neck cancer. Cell.

[84] Kadur Lakshminarasimha Murthy P. (2022). Human distal lung maps and lineage hierarchies reveal a bipotent progenitor. Nature.

[85] Xu Z. (2022). STOmicsDB: a database of spatial transcriptomic data. bioRxiv.

[86] Shay J.W., Wright W.E. (2005). Senescence and immortalization: role of telomeres and telomerase. Carcinogenesis.

[87] Akincilar S.C., Unal B., Tergaonkar V. (2016). Reactivation of telomerase in cancer. Cell Mol Life Sci.

[88] Cardoso-Moreira M. (2019). Gene expression across mammalian organ development. Nature.

[89] Leri A., Rota M., Pasqualini F.S., Goichberg P., Anversa P. (2015). Origin of cardiomyocytes in the adult heart. Circ Res.

[90] Cordero-Espinoza L., Huch M. (2018). The balancing act of the liver: tissue regeneration versus fibrosis. J Clin Invest.

[91] Lin S.D. (2018). Distributed hepatocytes expressing telomerase repopulate the liver in homeostasis and injury. Nature.

[92] Hathcock K.S., Chiang Y.J., Hodes R.J. (2005). regulation of telomerase activity and telomere length. Immunol Rev.

[93] Thorsson V. (2018). The immune landscape of cancer. Immunity.

[94] Chen B., Khodadoust M.S., Liu C.L., Newman A.M., Alizadeh A.A. (2018). Profiling tumor infiltrating immune cells with CIBERSORT. Cancer Systems Biology Methods Protocols.

[95] Lin J. (2016). Systematic and cell type-specific telomere length changes in subsets of lymphocytes. J Immunol Res.

[96] Patrick M., Weng N.-p (2019). Expression and regulation of telomerase in human T cell differentiation, activation, aging and diseases. Cell Immunol.

[97] Wang X.M. (2022). Comprehensive assessment of cellular senescence in the tumor microenvironment. Brief Bioinform.

[98] Correia-Melo C. (2016). Mitochondria are required for pro-ageing features of the senescent phenotype. EMBO J.

[99] Wiley C.D. (2016). Mitochondrial dysfunction induces senescence with a distinct secretory phenotype. Cell Metab.

[100] McCubrey J.A., LaHair M.M., Franklin R.A. (2006). Reactive oxygen species-induced activation of the MAP kinase signaling pathways. Antioxid Redox Signal.

[101] Son Y. (2011). Mitogen-activated protein kinases and reactive oxygen species: how can ROS activate MAPK pathways?. J Signal Transduct.

[102] Torres M., Forman H.J. (2003). Redox signaling and the MAP kinase pathways. Biofactors.

[103] Low K.C., Tergaonkar V. (2013). Telomerase: central regulator of all of the hallmarks of cancer. Trends Biochem Sci.

[104] Ackermann S. (2018). A mechanistic classification of clinical phenotypes in neuroblastoma. Science.

[105] Heaphy C.M. (2011). Prevalence of the alternative lengthening of telomeres telomere maintenance mechanism in human cancer subtypes. Am J Pathol.

[106] Ludlow A.T. (2014). Quantitative telomerase enzyme activity determination using droplet digital PCR with single cell resolution. Nucleic Acids Res.

[107] Hayflick L. (1965). The limited in vitro lifetime of human diploid cell strains. Exp Cell Res.

[108] Shay J.W., Bacchetti S. (1997). A survey of telomerase activity in human cancer. Eur J Cancer.

[109] Reddel R.R. (2003). Alternative lengthening of telomeres, telomerase, and cancer. Cancer Lett.

[110] Bryan T.M., Englezou A., Gupta J., Bacchetti S., Reddel R.R. (1995). Telomere elongation in immortal human-cells without detectable telomerase activity. Embo J.

[111] Li H.H., Li J., Zhang C.Y., Zhang C.X., Wang H.Y. (2020). TERT mutations correlate with higher TMB value and unique tumor microenvironment and may be a potential biomarker for anti-CTLA4 treatment. Cancer MedUs.

[112] Jafri M.A., Ansari S.A., Alqahtani M.H., Shay J.W. (2016). Roles of telomeres and telomerase in cancer, and advances in telomerase-targeted therapies. Genome Med.

[113] von Morgen P., Maciejowski J. (2018). The ins and outs of telomere crisis in cancer. Genome Med.

[114] Burbano M.S.J., Gilson E. (2020). Long-lived post-mitotic cell aging: is a telomere clock at play?. Mech Ageing Dev.

[115] Sullivan L.B., Santos J.H., Chandel N.S. (2012). Mitochondria and telomeres: the promiscuous roles of TIN2. Mol Cell.

[116] Haendeler J., Hoffmann J., Brandes R.P., Zeiher A.M., Dimmeler S. (2003). Hydrogen peroxide triggers nuclear export of telomerase reverse transcriptase via Src kinase family-dependent phosphorylation of tyrosine 707. Mol Cell Biol.

[117] Haendeler J., Hoffmann J., Rahman S., Zeiher A.M., Dimmeler S. (2003). Regulation of telomerase activity and anti-apoptotic function by protein-protein interaction and phosphorylation. Febs Lett.

[118] Minamino T., Kourembanas S. (2001). Mechanisms of telomerase induction during vascular smooth muscle cell proliferation. Circ Res.

[119] Cheng L. (2019). PES1 is a critical component of telomerase assembly and regulates cellular senescence. Sci Adv.

[120] Jose S.S., Bendickova K., Kepak T., Krenova Z., Fric J. (2017). Chronic inflammation in immune aging: role of pattern recognition receptor crosstalk with the telomere complex?. Front Immunol.

[121] Davalli P., Mitic T., Caporali A., Lauriola A., D’Arca D. (2016). ROS, cell senescence, and novel molecular mechanisms in aging and age-related diseases. Oxid Med Cell Longev.

[122] Liou G.-Y., Storz P. (2010). Reactive oxygen species in cancer. Free Radic Res.

[123] Billard P., Poncet D.A. (2019). Replication stress at telomeric and mitochondrial DNA: common origins and consequences on ageing. Int J Mol Sci.

[124] Santos J.H., Meyer J.N., Van Houten B. (2006). Mitochondrial localization of telomerase as a determinant for hydrogen peroxide-induced mitochondrial DNA damage and apoptosis. Hum Mol Genet.

[125] Reczek C.R., Chandel N.S. (2017). The two faces of reactive oxygen species in cancer. Annu Rev Cancer Biol.

[126] Dagg R.A. (2017). Extensive proliferation of human cancer cells with ever-shorter telomeres. Cell Rep.

[127] Panczyszyn A., Boniewska-Bernacka E., Goc A. (2020). The role of telomeres and telomerase in the senescence of postmitotic cells. DNA Repair.

[128] Han Y. (2025). Spatiotemporal analyses of the pan-cancer single-cell landscape reveal widespread profibrotic ecotypes associated with tumor immunity. Nat Cancer.

[129] Robinson M.D., McCarthy D.J., Smyth G.K. (2010). edgeR: a bioconductor package for differential expression analysis of digital gene expression data. Bioinformatics.

[130] Hänzelmann S., Castelo R., Guinney J. (2013). GSVA: gene set variation analysis for microarray and RNA-seq data. BMC Bioinforma.

[131] Noureen N., Ye Z., Chen Y., Wang X., Zheng S. (2022). Signature-scoring methods developed for bulk samples are not adequate for cancer single-cell RNA sequencing data. Elife.

[132] Noureen N., Wang X., Zheng S. (2022). Protocol to benchmark gene expression signature scoring techniques for single-cell RNA sequencing data in cancer. STAR Protoc.

[133] Aibar S. (2017). SCENIC: single-cell regulatory network inference and clustering. Nat Methods.

[134] Hao Y. (2021). Integrated analysis of multimodal single-cell data. Cell.

[135] Wang X., Langevin A.-M., Houghton P.J., Zheng S. (2022). Genomic disparities between cancers in adolescent and young adults and in older adults. Nat Commun.

