# Supplementary material for: Stratification of telomerase activity in cancer reveals associations with senescence and genomic instability
Source: Comput Struct Biotechnol J. 2025 Nov 14;27:5045–60. doi: 10.1016/j.csbj.2025.11.020 (PMC12663852; doi:10.1016/j.csbj.2025.11.020)
Supplement: Supplementary file 2 — Supplementary material [file mmc10.pdf]

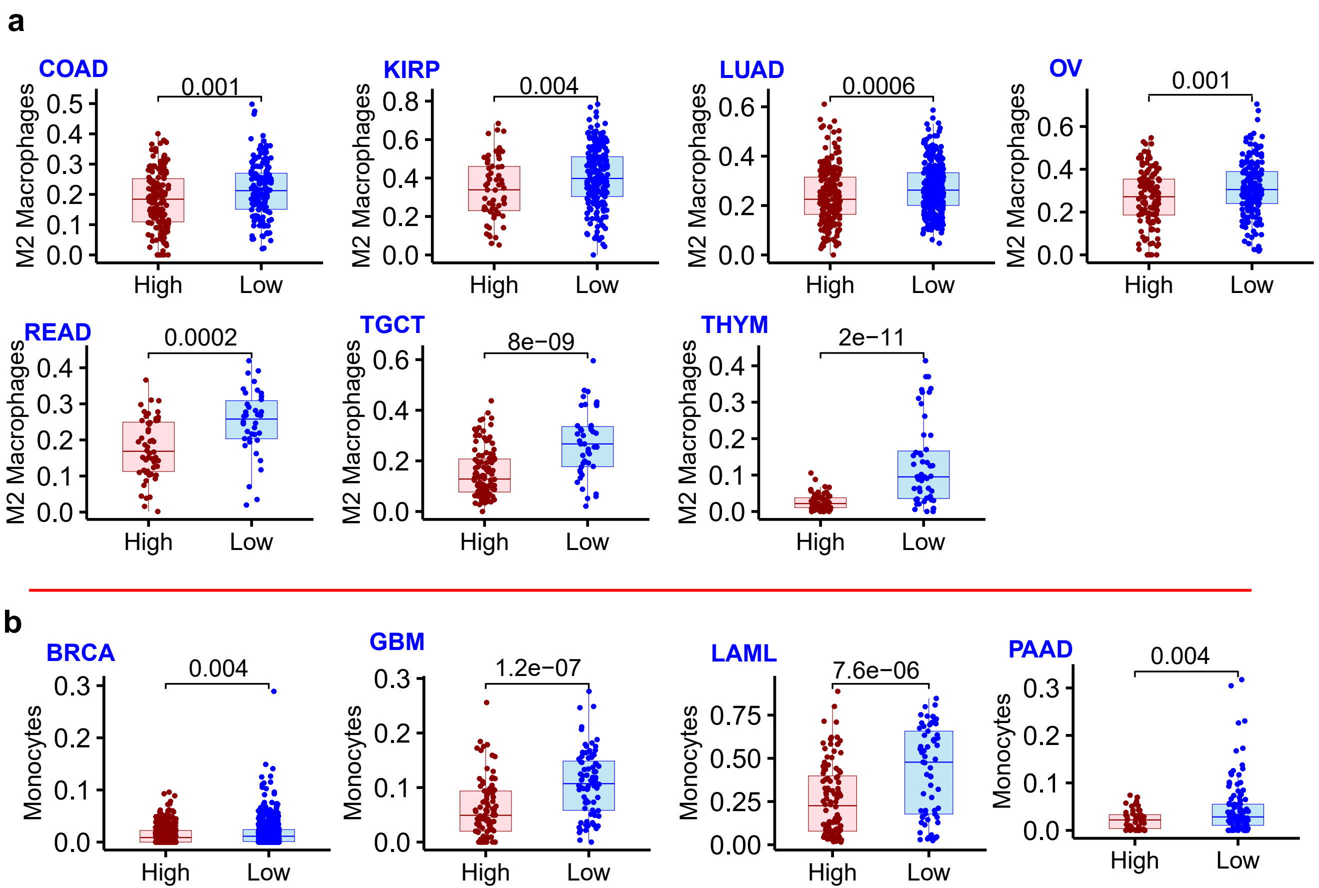

Supplementary Fig.9. Comparison of M2 macrophages and monocytes across telomerase activity groups (a) Differential M2 macrophage scores (y-axes) between low and high telomerase activity (EXTEND) groups (x-axes) across seven cancer types. (b) Comparison of monocyte scores (y-axes) between low and high telomerase activity groups (x-axes) across four cancer types. Cancer types with low telomerase activity dominance are labeled in blue. *P*-values were calculated using Student's *t*-test. Source data are available in the GitHub repository.
