# Supplementary material for: Stratification of telomerase activity in cancer reveals associations with senescence and genomic instability
Source: Comput Struct Biotechnol J. 2025 Nov 14;27:5045–60. doi: 10.1016/j.csbj.2025.11.020 (PMC12663852; doi:10.1016/j.csbj.2025.11.020)
Supplement: Supplementary file 3 — Supplementary material [file mmc11.pdf]

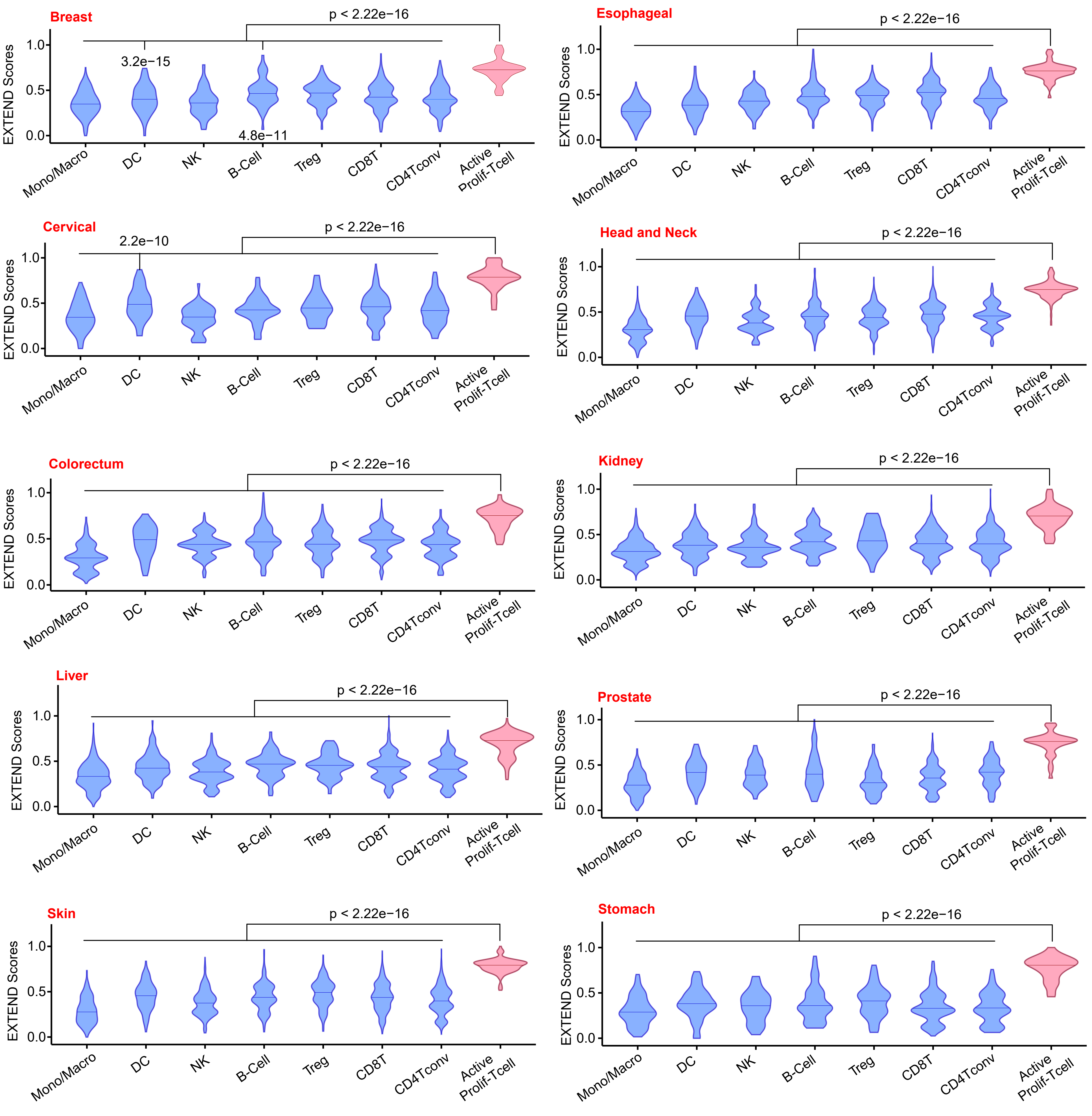

Supplementary Fig.10 Comparison of telomerase activity between proliferating and non-proliferating immune cells. Telomerase activity (EXTEND) scores are compared between single-cell RNAseq profiles of proliferating T cells (red) and non-proliferating immune cell populations (blue) across 10 cancer types. Cancer types are labeled in red. X-axes of violin plots represent immune cell populations,and Y-axes represent telomerase activity scores. The line in the middle of each violin plot represents the median. *P* values were calculated using Student’s *t*-test. Source data are available in the GitHub repository.
