# Supplementary material for: Stratification of telomerase activity in cancer reveals associations with senescence and genomic instability
Source: Comput Struct Biotechnol J. 2025 Nov 14;27:5045–60. doi: 10.1016/j.csbj.2025.11.020 (PMC12663852; doi:10.1016/j.csbj.2025.11.020)
Supplement: Supplementary file 4 — Supplementary material [file mmc2.pdf]

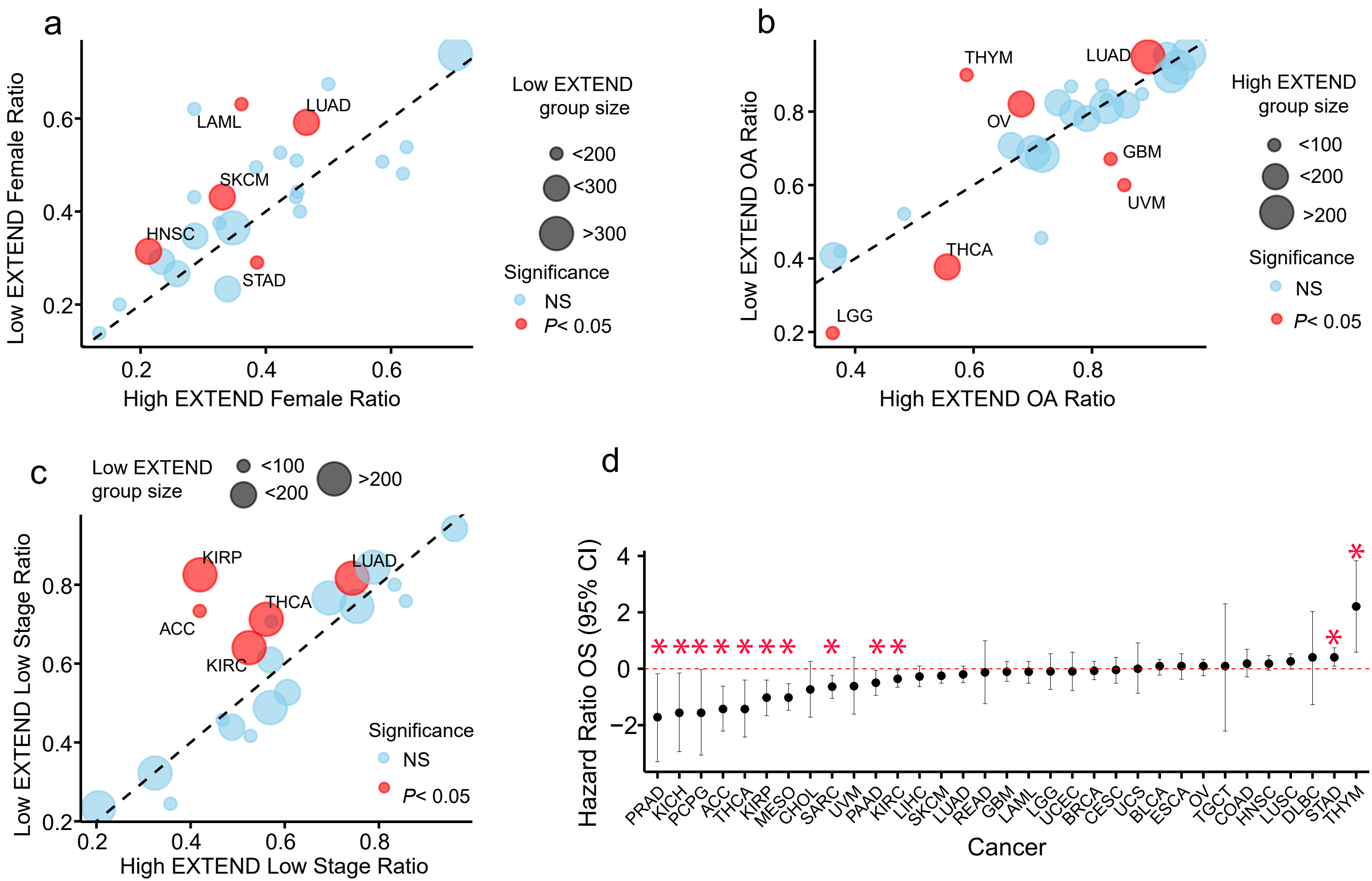

Supplementary Fig.1. Clinical disparities between low and high telomerase activity (EXTEND) groups across TCGA cancer types. **(a-c)** Comparisons of **(a)** female ratios, **(b)** older adult ratios, and **(c)** low stage ratios. Cancer types with significant differences ($P < 0.05$; Fisher's exact test) are labeled and highlighted in red, while non-significant (NS) cases are shown in blue.Circle size in **(a)** and **(c)** represents the number of samples in low telomerase activity group, and in **(b)** represents the number of samples in high telomerase activity group for each cancer type. X-axes represent ratios in high telomerase activity groups, and Y-axes show the corresponding ratios in low telomerase activity groups. **(d)** Hazard ratio (HR) plot from a univariate Cox regression model comparing low and high telomerase activity groups for 33 cancer types. Vertical bars indicate the 95% confidence interval limits of HR estimates. Significant cases (log-rank test; $P <= 0.05$) are marked with an asterisk. Source data are provided in the GitHub repository.
