# Supplementary material for: Stratification of telomerase activity in cancer reveals associations with senescence and genomic instability
Source: Comput Struct Biotechnol J. 2025 Nov 14;27:5045–60. doi: 10.1016/j.csbj.2025.11.020 (PMC12663852; doi:10.1016/j.csbj.2025.11.020)
Supplement: Supplementary file 5 — Supplementary material [file mmc3.pdf]

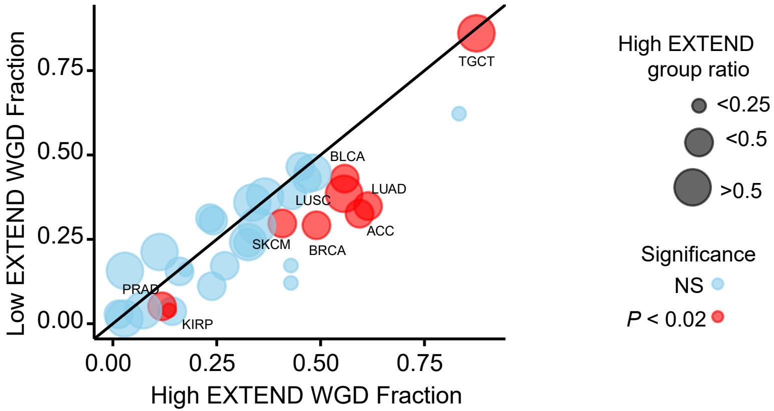

Supplementary Fig.2. Association of telomerase activity with whole genome doubling (WGD). Differential patterns of low and high telomerase activity (EXTEND) groups for WGD fractions across 33 TCGA cancer types. Cancer types with significant differences (*P*<0.02; Fisher's exact test) are labeled and highlighted in red, while non-significant (NS) cancer types are shown in blue. Circle size represents the number of samples in the high telomerase activity group for each cancer type. X and Y axes represent WGD fractions in high and low telomerase activity groups, respectively. Source data are provided in the GitHub respository.
