# Supplementary material for: Stratification of telomerase activity in cancer reveals associations with senescence and genomic instability
Source: Comput Struct Biotechnol J. 2025 Nov 14;27:5045–60. doi: 10.1016/j.csbj.2025.11.020 (PMC12663852; doi:10.1016/j.csbj.2025.11.020)
Supplement: Supplementary file 6 — Supplementary material [file mmc4.pdf]

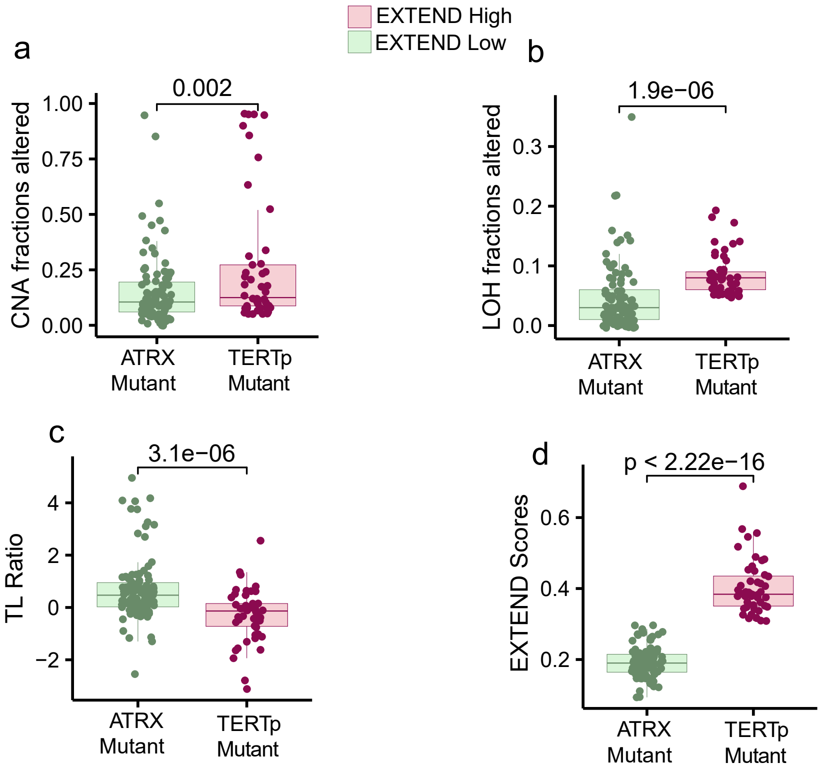

Supplementary Fig.3. Differential patterns between telomerase activity groups in low-grade glioma (LGG). (a-d) Comparisons of **(a)** Copy number altered fractions (CNA), **(b)** loss of heterozygosity (LOH), **(c)** telomere length ratios (TL), and **(d)** EXTEND scores between low and high telomerase (EXTEND) activity groups in LGG. The low telomerase group corresponds to ATRX-mutant cases (representing the ALT phenotype), while the high telomerase activity group corresponds to the TERT promoter-mutant cases (representing telomerase activation). Y-axes across panels (a-d) represent the respective metrics across two telomerase activity groups. X-axes across panels (a-d) represent the telomerase activity groups mapped to ATRX mutant and TERT promoter mutant cases in LGG. Source data are available in the GitHub repository.
