# Supplementary material for: Stratification of telomerase activity in cancer reveals associations with senescence and genomic instability
Source: Comput Struct Biotechnol J. 2025 Nov 14;27:5045–60. doi: 10.1016/j.csbj.2025.11.020 (PMC12663852; doi:10.1016/j.csbj.2025.11.020)
Supplement: Supplementary file 7 — Supplementary material [file mmc5.pdf]

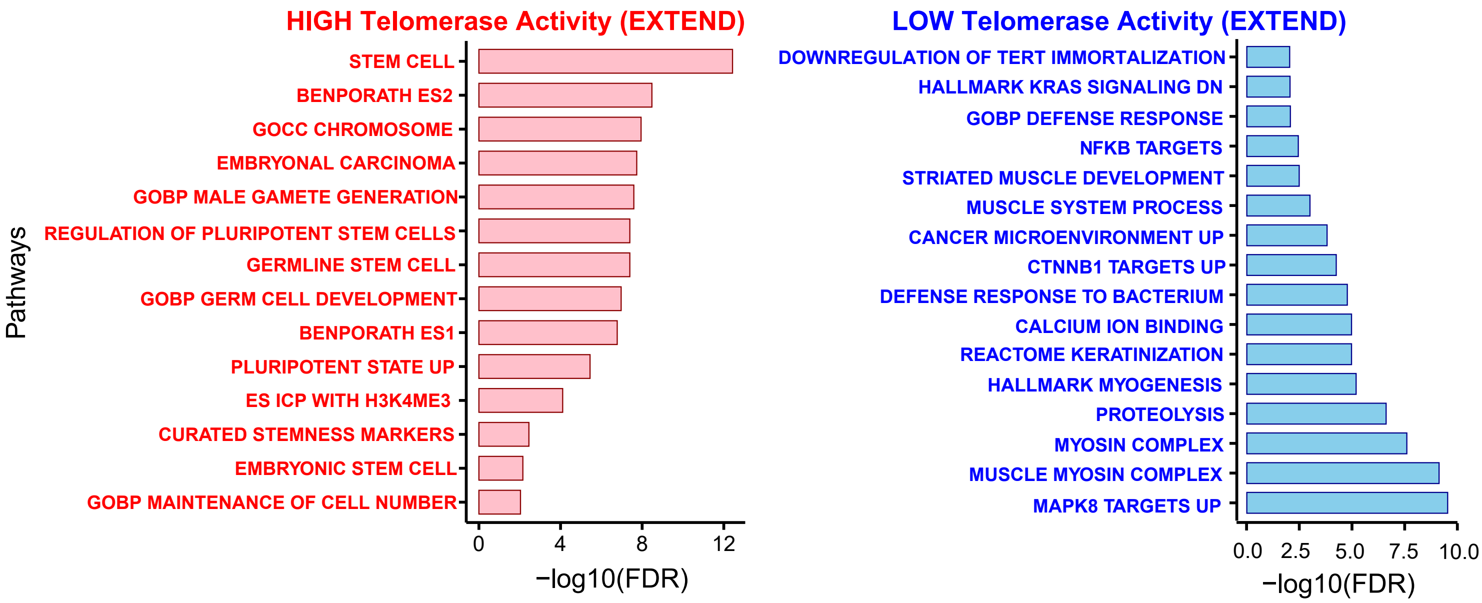

Supplementary Fig.4. Pathway enrichment associated with telomerase activity groups. Upregulated pathways in high and low telomerase activity (EXTEND) groups across TCGA pan-cancer data (33 Cancer types). The X-axes represent the significance level (-log10 FDR).Y-axes represent the enriched pathways. Blue color indicates pathways upregulated in the low telomerase activity group, while red color indicates upregulated pathways in the high telomerase activity group. Source data are available in the GitHub repository.
