# Supplementary material for: Stratification of telomerase activity in cancer reveals associations with senescence and genomic instability
Source: Comput Struct Biotechnol J. 2025 Nov 14;27:5045–60. doi: 10.1016/j.csbj.2025.11.020 (PMC12663852; doi:10.1016/j.csbj.2025.11.020)
Supplement: Supplementary file 8 — Supplementary material [file mmc6.pdf]

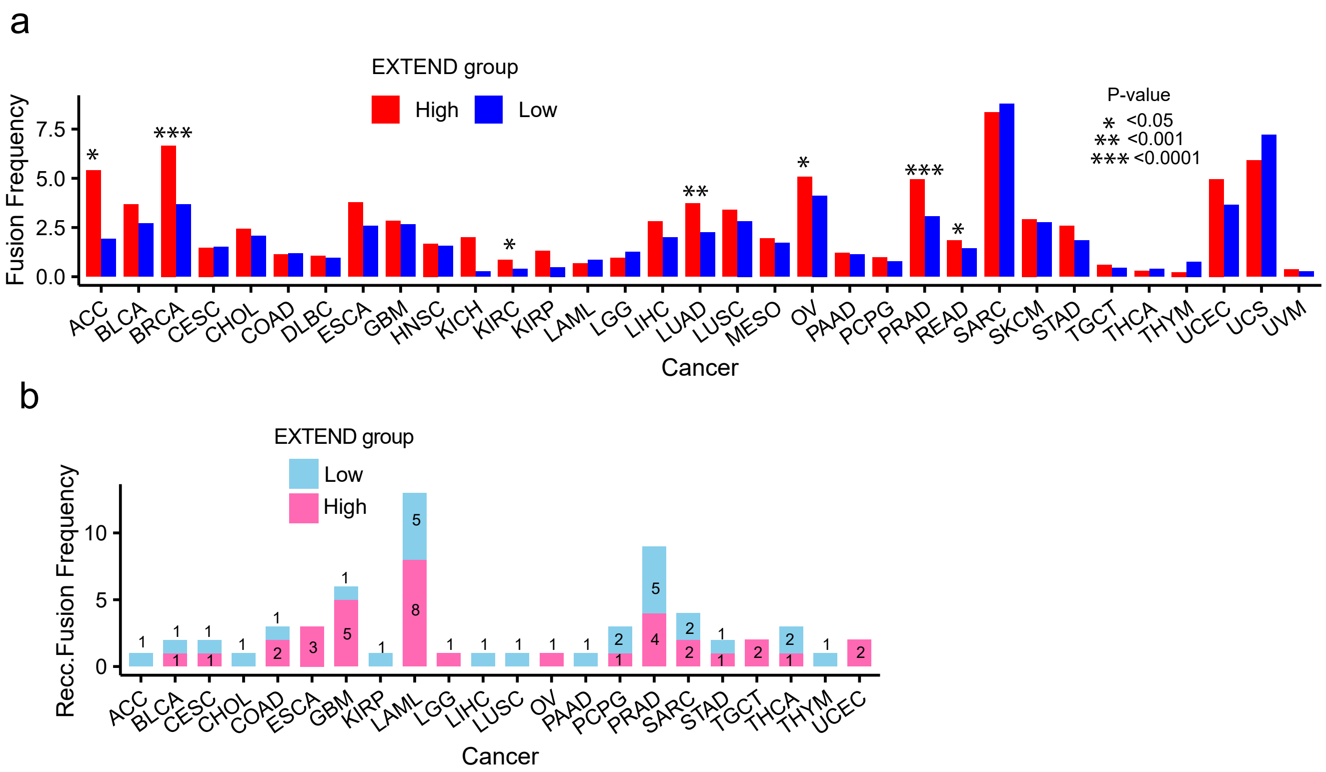

Supplementary Fig.5. Differential gene fusion frequencies across telomerase activity groups. (a) Comparison of gene fusion frequencies (y-axis) between low (blue) and high (red) telomerase activity (EXTEND) groups acrossTCGA pan-cancer data (x-axis). Significant cases (Student's *t*-test ; $P < 0.05$) are indicated with asterisk(*). **(b)** Recurrent fusion frequencies (y-axis) in low (blue) and high (pink) telomerase activity groups across the pan-cancer cohort. Numbers on each bar indicate the total number of gene pairs for each case. Only fusions present in more than 1% of cases per cancer type (x-axis) were included in the analysis. Source data are available in the GitHub repository.
