# Supplementary material for: Stratification of telomerase activity in cancer reveals associations with senescence and genomic instability
Source: Comput Struct Biotechnol J. 2025 Nov 14;27:5045–60. doi: 10.1016/j.csbj.2025.11.020 (PMC12663852; doi:10.1016/j.csbj.2025.11.020)
Supplement: Supplementary file 9 — Supplementary material [file mmc7.pdf]

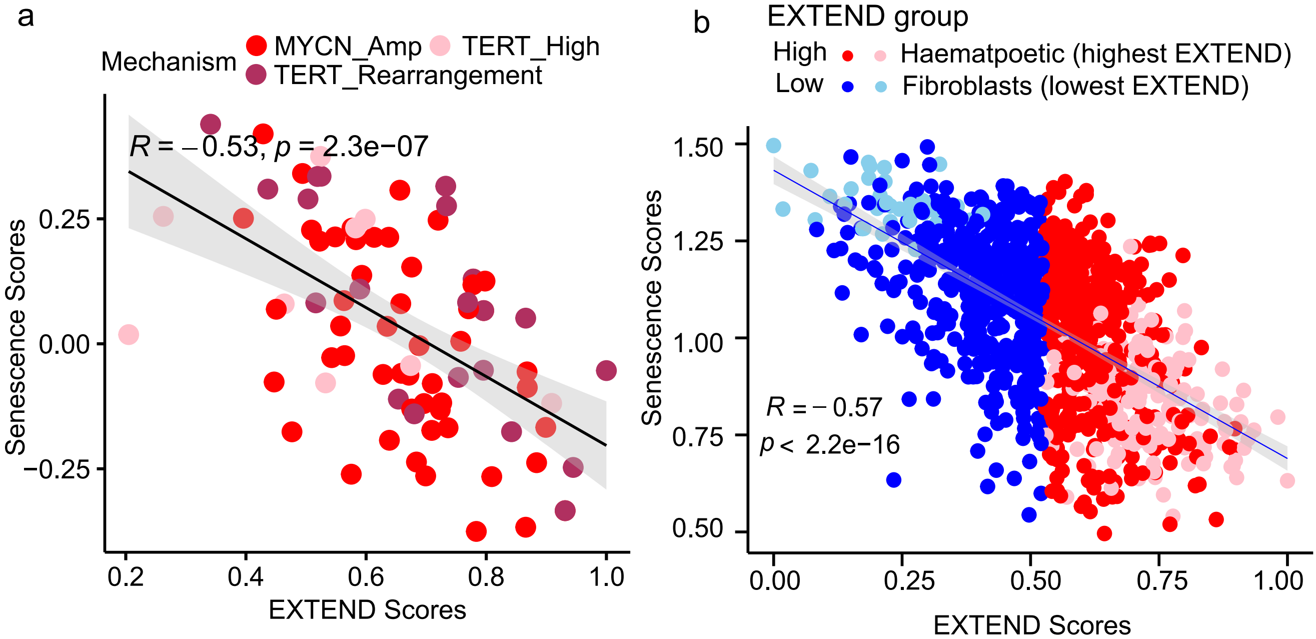

Supplementary Fig.6. Association of telomerase activity with senescence. Spearman correlations between telomerase activity (EXTEND) scores and senescence scores in **(a)** neuroblastoma data (Ackerman *et al*., 2018) and **(b)** Cancer Cell line Encylopedia **(**CCLE) data. X-axes represent telomerase activity scores, and Y-axes represent senescence scores in both panels. In (a) color coding represents telomerase activity subgroups in neuroblastoma data. In (b) colors indicate low and high telomerase activity groups, the lowest (light blue = fibroblasts) and highest (pink = hematopoietic) cell lines are marked separately, while remaining low and high telomerase activity cell lines are shown in blue and red respectively. Source data are available in the GitHub repository.
