# Supplementary material for: Stratification of telomerase activity in cancer reveals associations with senescence and genomic instability
Source: Comput Struct Biotechnol J. 2025 Nov 14;27:5045–60. doi: 10.1016/j.csbj.2025.11.020 (PMC12663852; doi:10.1016/j.csbj.2025.11.020)
Supplement: Supplementary file 10 — Supplementary material [file mmc8.pdf]

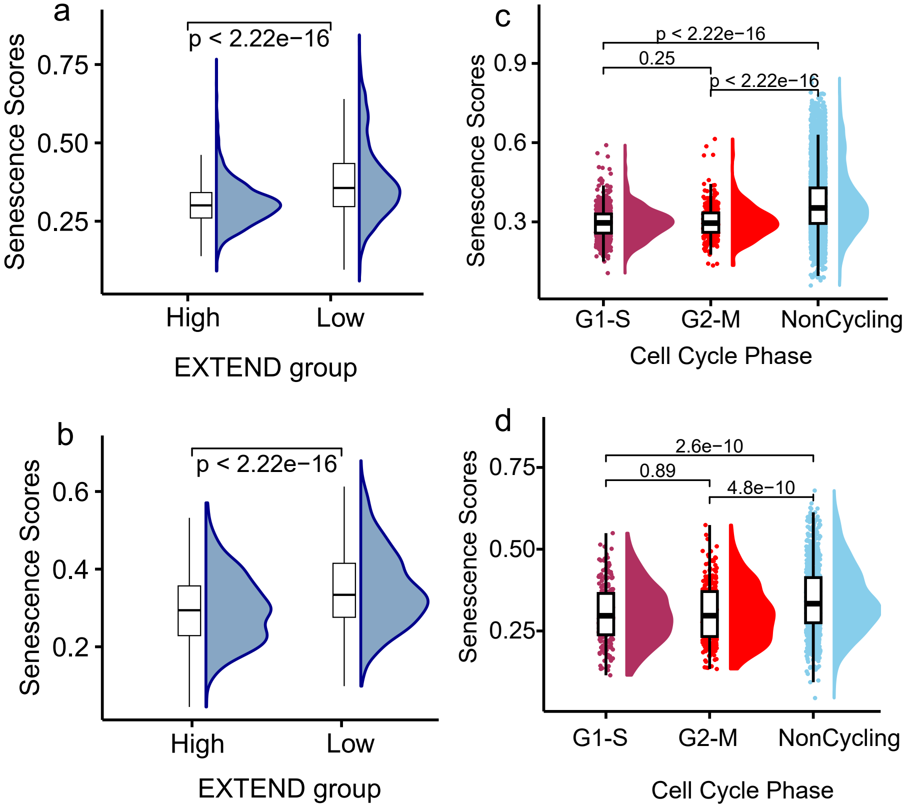

Supplementary Fig.7. Comparison of senescence scores in single-cell data. Differential senescence patterns between low and high telomerase activity (EXTEND) groups in single-cell datasets from **(a)** GBM and **(b)** HNSC. Distribution of senescence scores across cell-cycle phases in **(c)** GBM and **(d)** HNSC datasets. *P* values were calculated using Student's *t*-test. Y-axes represent senescence scores in panels (a-d) and X-axes represent telomerase activity groups in (a-b) and cell-cycle phases in (c-d). Source data is available in the GitHub repository.
