# Supplementary material for: Stratification of telomerase activity in cancer reveals associations with senescence and genomic instability
Source: Comput Struct Biotechnol J. 2025 Nov 14;27:5045–60. doi: 10.1016/j.csbj.2025.11.020 (PMC12663852; doi:10.1016/j.csbj.2025.11.020)
Supplement: Supplementary file 11 — Supplementary material [file mmc9.pdf]

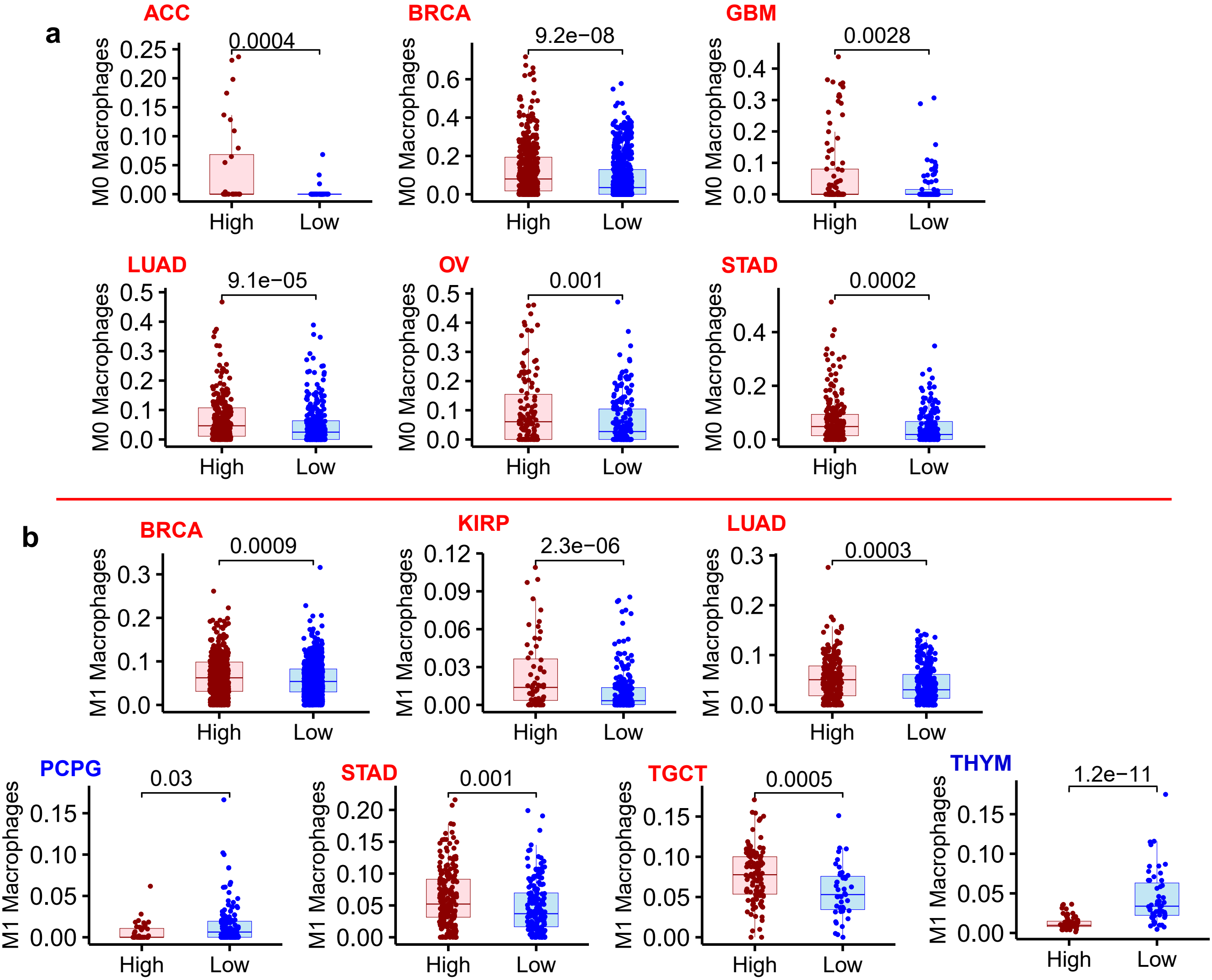

Supplementary Fig.8. Comparison of M0 and M1 macrophages across telomerase activity groups (a) Differential M0 macrophage scores (y-axes) between low and high telomerase activity (EXTEND) groups (x-axes) across six cancer types. (b) Comparison of M1 macrophage scores (y-axes) between low and high telomerase activity groups (x-axes) across seven cancer types. Cancer types with high telomerase activity dominance are labeled in red, and those with low telomerase activity dominance are labeled in blue. *P* values are calculated using Student's *t*-test. Source data is available in GitHub repository.
